# *Hibiscus rosa‐sinensis* : A Multifunctional Flower Bridging Nutrition, Medicine, and Molecular Therapeutics

**DOI:** 10.1002/fsn3.71254

**Published:** 2025-11-27

**Authors:** Hassan Raza, Muhammad Tauseef Sultan, Khalil Ahmad, Muhammad Maaz, Shehnshah Zafar, Ahmad Mujtaba Noman, Entessar Mohammad Al Jbawi

**Affiliations:** ^1^ Department of Human Nutrition, Faculty of Food Science and Nutrition Bahauddin Zakariya University Multan Pakistan; ^2^ Department of Chemistry Emerson University Multan Multan Pakistan; ^3^ Sugar Beet Research Department, Crop Research Administration General Commission for Scientific Agricultural Research (GCSAR) Damascus Syria

**Keywords:** antioxidant, *Hibiscus rosa‐sinensis*, molecular docking, phytochemistry

## Abstract

*Hibiscus rosa‐sinensis* , commonly known as the shoe flower, thrives in tropical and subtropical regions across South China, Asia, Africa, and America, adapting well to diverse environments. Its therapeutic potential is attributed to a range of bioactive compounds Anthocyanins, saponins, quercetin, kaempferol, and anthraquinones are found in various plant parts. These compounds scavenge reactive oxygen and nitrogen species and modulate inflammatory (IL‐6, TNF‐α, PPAR‐γ, cyclooxygenase) and oxidative (MDA, MPO, NO, SOD, GSH) markers, offering therapeutic benefits against cancer, diabetes, cardiovascular, neurological, gastrointestinal, and hepato‐renal disorders. The plant exhibits antidiabetic effects by lowering blood glucose, triglycerides, cholesterol, and postprandial glucose absorption. Cardioprotective properties are linked to the regulation of PPAR‐γ, SREBP‐1c, acetyl‐CoA carboxylase, and fatty acid synthase. Molecular docking studies reveal strong binding affinities of myricetin and rutin with α‐glucosidase (−10.5) and SOD (−8.6), highlighting their antidiabetic and hepatoprotective potential. *H. rosa‐sinensis* also exhibits antimicrobial activity against various bacterial (e.g., *E. coli* , *S. aureus* ) and fungal strains (e.g., *A. flavus* , *A. niger* ). Animal‐based studies affirm its safety between 400 and 800 mg/kg body weight. Its coloring, flavoring, nutritional, and therapeutic properties support applications in food, cosmetics, nutraceuticals, and dyeing. However, long‐term clinical trials are essential to validate its traditional uses and therapeutic efficacy.

## Graphical abstract

*H. rosa‐sinensis* bioactives modulate oxidative and inflammatory pathways. Myricetin and rutin show strong α‐glucosidase and SOD binding affinity. Exhibits safe, multifunctional use in health, food and nutraceuticals.

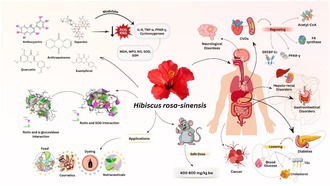

**Abbreviations**
ABTS: 2,2'‐azino‐bis‐(3‐ethylbenzothiazoline‐6‐sulfonic) acid
AIDS: acquired immunodeficiency syndrome
ALP: alkaline phosphatase
ALT: alanine transaminase
AMPK: AMP‐activated protein kinase
AST: aspartate transferase
C3: carbon 3
CAT: catalase
CCL4: carbon tetrachloride
CD4: cluster of differentiation 4
CMC: carboxy methyl cellulose
CuO NPs: copper oxide nanoparticles
DPPH: 2,2‐diphenyl‐1‐picrylhydrazyl
ESR1: estrogen receptor 1
FRAP: ferric reducing antioxidant power
GABA: gamma‐Aminobutyric acid
GAE: gallic acid equivalents
GSH: glutathione
HCT: hematocrit
HDL: high density lipoprotein
HER2: human epidermal growth factor receptor 2
HIV: human immunodeficiency virus
IgE: immunoglobulin E
IL‐6: interleukin 6
LD: lethal dose
LDL: low density lipoprotein
MCF: Michigan Cancer Foundation
MDA: malondialdehyde
MIC: minimum inhibitory concentration
MPO: myeloperoxidase
MTT: 3‐ (4,5‐dimethylthiazol‐2‐yl)‐2,5‐diphenyltetrazolium bromide
NO: nitric oxide
PDB: protein data bank
PPAR‐γ: peroxisome proliferator‐activated receptor gamma
PRISMA: Preferred Reporting Items for Systematic reviews and Meta‐Analyses
ROS's: reactive oxygen species
SOD: superoxide dismutase
SREBP‐1c: Sterol regulatory element binding protein‐1c
TC: total cholesterol
TFC: total flavonoid content
TG: triglyceride
TNF‐α: tumor necrosis factor alpha
TPC: total phenolic content
VLDL: very low‐density lipoprotein

## 1 Introduction

The medicinal herbs and flowers have changed the aura of the 21st century and shifted global trends towards natural herbal remedies due to their pharmacological aspects. The development of ethnomedicine and nutraceuticals has significantly impacted humanity and plays a key role in health promotion (Chaachouay and Zidane 2024). Multiple communities from ancient times have used plants for various purposes, including food, shelter, and earning sources, based on their knowledge, skills, and beliefs (Zemede et al. 2024). Moreover, these flowers and herbs are the backbone of traditional healthcare systems and have been used for centuries to cure several acute and chronic disorders (Ralte et al. 2024). Despite modern commercialized synthetic drugs that are available in the market and have immediate responses to manage the symptoms of diseases, more than 80% of the current world population uses medicinal plants as primary treatment sources (Yadav et al. 2024).

Several adverse health consequences, such as gastrointestinal disturbance, neurological impairments, and heart and renal failure, have been reported from the utilization of synthetic drugs (Rehman et al. 2024). According to the World Health Organization, ~5 million individuals die each year due to improper and false drug recommendations. These figures demanded the formulation of such drugs, which have limited adverse health effects and better health benefits (Ahmed et al. 2024). Nutraceutical and food industrialists have developed nutraceuticals and functional foods that modulate the normal physiology of the human body (Keservani et al. 2024; Shi et al. 2024).

Medicinal plants and their compounds are safe to consume with multiple health‐promoting and disease‐reducing properties. *H. rosa‐sinensis* , an evergreen herbaceous medicinal plant, belongs to the Malvaceae family and Hibiscus genus that has around 275 species in tropical and subtropical regions of the world (Raza et al. 2025; Geeganage and Gunathilaka 2024). *H. rosa‐sinensis* is renowned for its diverse nomenclature across the world's various regions. It is Gul‐e‐Gurhal in Pakistan, Chembarathi in Malayalam, Semparutti in Tamil, Rudrapuspa in Sanskrit, Gurhal in Hindi, Shoe flower plant and Chinese hibiscus in English (Sivaraman and Saju 2021). Flavonoids and polyphenolic compounds present in *H. rosa‐sinensis* are responsible for its anti‐inflammatory, antitumorigenic, antibacterial, antifungal, anti‐diabetic, antioxidant, and antipyretic properties (Sanadheera et al. 2021).

This multidimensional review focuses on the nutritional composition and phytochemistry of *H. rosa‐sinensis* . It effectively conveys that the content goes far beyond ornamental uses, highlighting *H. rosa‐sinensis's* medicinal properties, safety concerns, and the industrial value of different parts. Moreover, the studies on the synergy of *H. rosa‐sinensis* with other medicinal plants are the highlight of this article. The combination of health, safety, and economic perspectives ensures relevance across disciplines, making this review unique and impactful. Lastly, this review is the first to comprehensively report molecular docking regarding hepatoprotective and antidiabetic roles, offering new insights into the therapeutic potential of *H. rosa‐sinensis*.

## 2 Methodology

**FIGURE 1:**
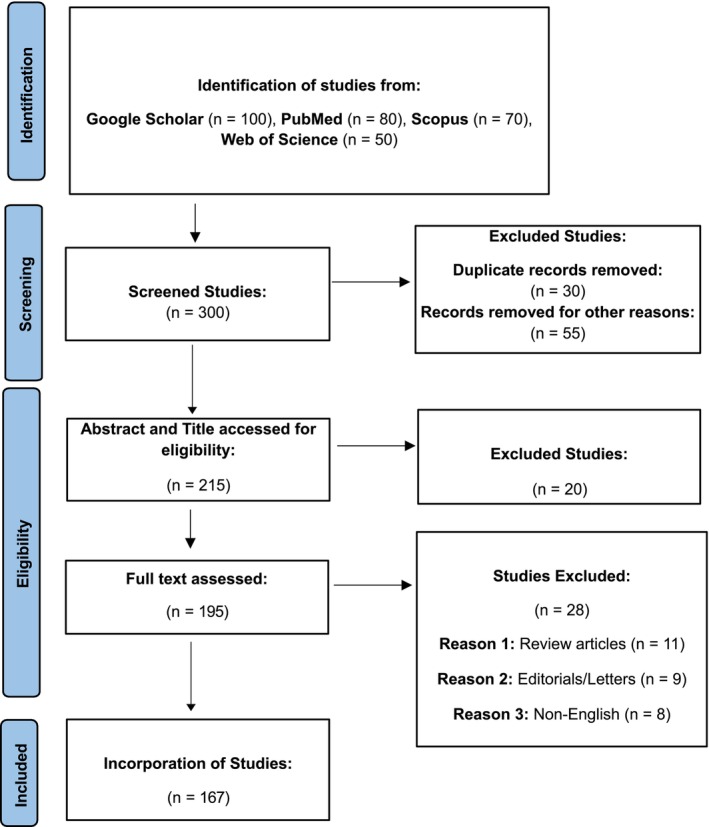
PRISMA flow diagram of the literature search process.

As shown in the PRISMA flow diagram (Figure 1), 300 studies were found. After removing duplicates and excluding irrelevant papers based on titles and abstracts, 195 articles were retained for full‐text review. Fifteen articles were excluded after full‐text review, with the reasons for their removal detailed in the PRISMA flow diagram. Finally, 180 studies were included in the review.

## 3 Taxonomical Classification

**Table:** 

**Kingdom**	**Plantae**
Subkingdom	Tracheobionta
Division	Magnoliophyta
Class	Magnoliopsida
Subclass	Dilleniidae
Order	Malvaceae
Genus	*Hibiscus*
Species	*H. rosa‐sinensis*

(Iqbal and Rehman 2023).

## 4 Geographical Distribution

*H. rosa‐sinensis* is an herbal flowering plant distributed across the world's tropical and subtropical regions, particularly in South China, tropical Asia, Africa, and America, which provides favorable conditions for its growth and germination. However, its growth depends on soil fertility, nutrient availability, irrigation system, and environmental and climatic conditions (Magdalita and San Pascual 2022; Shah and Wu 2019).

## 5 Botanical and Morphological Description

**FIGURE 2:**
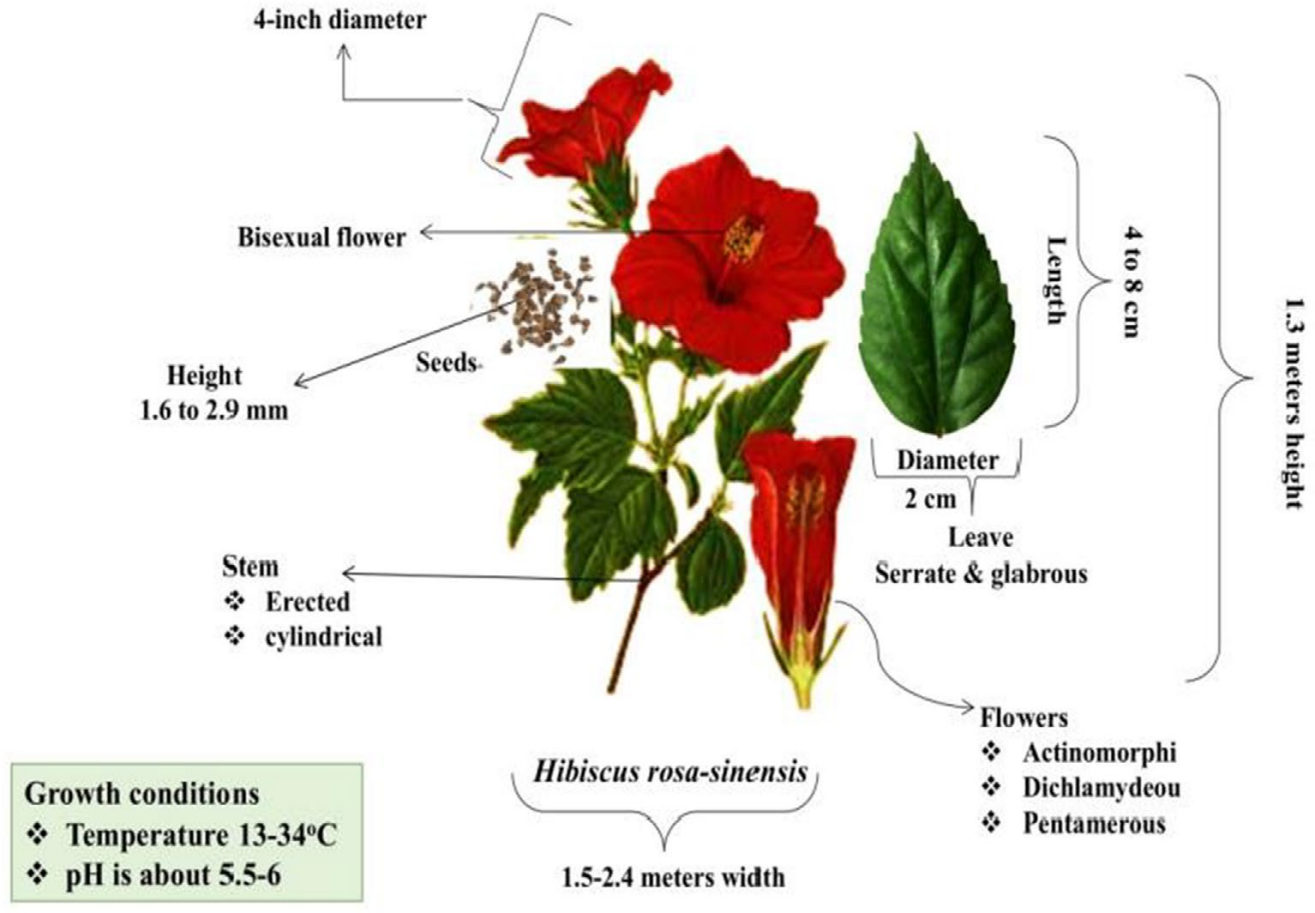
Botanical description of *Hibiscus rosa‐sinensis*.

*H. rosa‐sinensis* is a perennial shrub that grows to a height of 1.3 m with a width of 1.5–2.4 m. Its dull green leaves are simple, serrate, and glabrous, with a length of 4 to 8 cm and a width of 2 cm. Its flowers are five‐petalled with a 4‐in. diameter and are bisexual, showy, actinomorphic, dichlamydeous, and pentamerous. The seeds aid in the germination process of *H. rosa‐sinensis* , which occurs because of pollination in appropriate environmental conditions. It has abortive fruit, erect and cylindrical stems, and branched tap roots, while its dark brown seeds are 1.6 to 2.9 mm tall. Its growth is at its peak when the temperature is optimum, i.e., 13°C–34°C, and pH is about 5.5–6. Its cultivation is significantly increased due to its pest, disease, and drought‐resistant properties (Shaheen et al. 2023; Valdivié and Martínez 2022). The botanical and morphological description of *H. rosa‐sinensis* is demonstrated in Figure 2.

## 6 Nutritional Composition

The nutritional profile of *H. rosa‐sinensis* significantly contributes to improving healthy lifestyles and overall well‐being. Different factors such as biofortification, enrichment, and fermentation can improve the nutritional profile and enhance the bioavailability of nutrients within the body (Moyo 2024). Studies have reported that nutrient content can vary depending on environmental factors. It has been found that the moisture (2.6%–7.7%), ash (3%–14%), fat (2%–9%), fiber (3.1%–3.9%), proteins (2%–7%), and carbohydrate contents are (32%–73%), respectively in the leaves of *H. rosa‐sinensis* (Eze and Nwibo 2017; Udo et al. 2016). However, the flowers of *H. rosa‐sinensis* have moisture (76%–83%), ash (5.5%–6.3%), fats (0.3%–1.2%), fiber (1.5%–2%), proteins (1.54%–2.4%), and carbohydrates (13%–15%) (Bala et al. 2022; Al‐Snafi 2018). Vitamins such as vitamin A (2.5), vitamin B2 (1.5), vitamin C (20), vitamin E (10 mg/100 g) and minerals including potassium (5.4), iron (9.5), calcium (9.3 mg/100 g), phosphorous (42 mg/g‐1), sodium (0.4 mg/g‐1), magnesium (90 mg/g‐1), and manganese (2.4 mg/g‐1) are the main micro‐constituents of dried leaves of *H. rosa‐sinensis* (Eze and Nwibo 2017; Udo et al. 2016).

## 7 Phytochemical Composition

*H. rosa‐sinensis* contains several bioactive compounds that impose multiple therapeutic attributes and contribute to improving individuals' health. Shafiq et al. (2021) identified caffeic acid, gallic acid, and p‐coumaric acid at 15, 11, and 35 ppm concentrations. Furthermore, the bioactive compounds in the roots are (flavonoids, tannins, glycosides, resins, reducing sugars, saponins, gums and mucilage) (Amtaghri et al. 2024), leaves (alkaloids, quercetin, kaempferol, anthocyanins, sterols, glycosides, and fats) (Umar et al. 2024), stems (sitosterol, cardiac glycosides, anthraquinones, malvalic acids and cyclic sterculic acid) (Tawfeeq et al. 2024), and flowers (thiamine, riboflavin, niacin, apigenidine, oxalic, citric and ascorbic acids) (Zulkurnain et al. 2023; Bala et al. 2022).

The essential oil extracted from the flowers contains bioactive compounds such as 1‐iodoundecane, 1, 2‐benzenedicarboxylic acid isodecyl octyl ester, neopentane, 2‐propenamide, 1–4 butanediol ester, 2, 2, 4‐trimethyl 3‐pentanone, 2‐cyclopentylethanol, 1‐tetrazole‐2‐ylethanone, amyl nitrite, and 4‐trifluoroacetoxyoctane (Agarwal and Prakash 2013). The essential oil of *H. rosa‐sinensis* leaves comprises limonene, carvone, tetradecane, (E)‐caryophyllene, 2‐ethylhexyl mercaptoacetate, bisabolene, caryophyllene oxide, 1,3,7,11‐cyclotetradecatetraene, dibutyl phthalate, docosyl heptafluorobutyrate, and squalene, performing multiple functions within the body systems (Sidhu et al. 2023). The chemical structures of *H. rosa‐sinensis* bioactive compounds are drawn below.


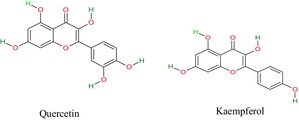
 
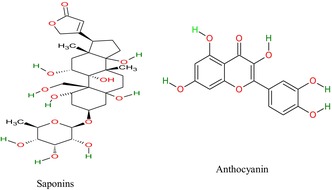


## 8 Traditional Applications

*H. rosa‐sinensis* has been traditionally used in primary healthcare systems to cure multiple disorders. Healthcare professionals formulate different decoctions, amalgams, and creams using parts of *H. rosa‐sinensis* against specific ailments. These formulations vary from region to region. Bangladesh prepares decoctions by using the flowers of *H. rosa‐sinensis* to modulate the regular menstrual cycles. At the same time, the Chinese formulate hot water extract from their flowers and bark to regulate menstrual periods and reduce menstrual pain. In addition to menstruation regulation, *H. rosa‐sinensis* also contains other traditional health implementations (Bala et al. 2022). The hot water extracts of its dried leaves and flowers are used to treat sick infants, gonorrhea, influenza, dry and productive cough, stimulate labor during delivery, and bronchitis. Its roots and bark are beneficial in the management of sexually transmitted disorders (HIV and AIDS), cough, skin disorders, and menorrhagia (Amtaghri et al. 2024; Magdalita and San Pascual 2022; De Boer and Cotingting 2014). Table 1 includes the traditional uses of *H. rosa‐sinensis* different parts in various regions of the world (Bala et al. 2022).

**TABLE 1:** Traditional uses of *H. rosa‐sinensis* different parts in different forms throughout the world.

Parts	Form/route	Uses/benefits	Country
Flower	Hot water extract	Emmenagogue	China
Leaves and flowers		Sick infants	Cook Islands
Leaves and flowers		Gonorrhea	Cook Islands
Flowers		Reproductive issues	East indies
Flowers		Grippe	French Guiana
Root and bark		External emollients	Philippines
Flowers		Regulate menstruation	New Britain (East)
Dried stems		Contraceptive and emmenagogue	Peru
Flowers and leaves		Ease childbirth	Fiji
Leaves	Fresh juice	Diarrhea and improves reproductivity	Fiji
Fresh leaves	Decoction	Antidiarrheal	Japan
Flowers	Orally	Emmenagogue and aphrodisiac	Kuwait
Roselle juice	Juice	To quench thirst	Thailand
Bark	Extract	Emmenagogue	Vietnam
Bark, stem	Decoction	Menorrhagia	Vanuata
Leaves	Eaten as spinach	Food	Nyasaland in South Africa
Fresh flowers	Infusion (orally)	Abortifacient	Rarotonga
Flowers and leaves	Water extract	Induce labor	Samoa
Young leaves	Water (orally)	Induce labor	Ireland
Flower and bark	Infusions	Treatment of dysentery	Mexico

## 9 Medicinal Properties

*H. rosa‐sinensis* has various health‐promoting and disease‐ameliorating properties, which are mainly due to the presence of bioactive constituents. These components aid in alleviating the progression and severity of different health disorders such as diabetes, cardiovascular disorders, liver cirrhosis, and arthritis.

### 9.1 Antioxidant Potential

The antioxidant potential of plants is their ability to scavenge and neutralize ROS, thus helping reduce oxidative stress and inflammation and maintaining homeostasis in the body (Goyal et al. 2025). Although synthetic antioxidants have been prepared, due to their low effectiveness, natural antioxidants are frequently used to reduce free radicals (Uzombah 2022). The capacity of *H. rosa‐sinensis* to scavenge and neutralize ROS makes it a suitable option as a natural antioxidant. Various studies have been conducted to validate the antioxidant properties of *H. rosa‐sinensis*. The antioxidant activity of *H. rosa‐sinensis'* isolated compounds (C3, C4, and C5) and vitamin C was evaluated by determining ABTS scavenging activity, lipid peroxidation scavenging activity, metal chelating activity, nitric oxide scavenging activity, superoxide scavenging activity, and hydrogen peroxide scavenging activity which demonstrated that compound C5 has higher free radical scavenging activity as compared to C3, C4, and ascorbic acid. The respective EC50 values were 2.82, 4.70, 5.22, 6.57, 8.10, and 5.19 μg/mL (Rengarajan et al. 2020). Earlier, Thi et al. (2021) revealed the antioxidant potential of *H. rosa‐sinensis* by conducting a DPPH assay at varying concentrations of 0.05%, 0.1%, 0.15%, 0.2%, 0.25%, and 0.3%. The results depicted the maximum DPPH free radical's inhibitory effect (87.42%) at 0.3% concentration compared to other concentrations.

Moreover, Sidhu et al. (2023) conducted DPPH, NO, and ABTS assays to assess the antioxidant potential of *H. rosa‐sinensis* leaves essential oil and dibutyl phthalate. It was observed that the essential oil has more antioxidant potential with IC50 values of 1240 μg/mL (DPPH), 1300 μg/mL (NO), and 1460 μg/mL (ABTS). The DPPH assay revealed that the methanolic extract of *H. rosa‐sinensis* leaf and flowers was revealed by Sharma and Thakur (2022) with respective IC50 values of 98 and 117 μg/mL. Similarly, its ethanolic leaf extract showed free radical scavenging potential as determined by the DPPH method with the IC50 value of 18.70 μg/mL (Kumar Dwivedi and Jain 2023). Ghaffar and El‐Elaimy (2012) conducted total antioxidant activity and reducing power assays, which revealed that *H. rosa‐sinensis* extract has a significant free radical scavenging effect compared to butylated hydroxytoluene at 500 μg/mL. Furthermore, Khan et al. (2014) demonstrated the free radical scavenging potential of *H. rosa‐sinensis* flowers, which showed DPPH inhibition of 75.4% and 64.9% by its methanol and ethanol extracts, respectively.

Additionally, Falade et al. (2009) indicated DPPH inhibition by the methanolic extract of flowers, which was 43.9 mg/mL. The observed DPPH inhibition was 83% and 97% by the ethanol and aqueous extract of *Hibiscus* flower (Mak et al. 2013). Chai (2020) evaluated the free‐radical scavenging potential of white, orange, and hybrid hibiscus flowers and leaves by conducting DPPH, TPC, TFC, and FRAP assays. It has been shown that orange flowers have the highest antioxidant potential (90%) as compared to white (81%) and hybrid (75%) *Hibiscus*, with the respective values of DPPH (90.45%), TPC (33 mg GAE/g), and FRAP (125.38 mg FE/g). Similarly, Kumar Dwivedi and Jain (2023) revealed that the ethanol extract of hibiscus leaves has the highest antioxidant potential with an IC50 value of 18.70 μg/mL.

### 9.2 Anticancer Activity

Cancer is a proliferative disorder that rapidly spreads and disrupts the normal growth of cells. Cancer prevalence constantly increases with advancements in food, environment, industries, and healthcare facilities (Johariya et al. 2024). According to an estimation, approximately 20 million cases of cancer were reported in 2024, with 9.7 million fatalities. These figures demand effective management strategies with limited toxic effects to reduce mortality and cancer prevalence (Noman et al. 2025). Researchers have employed a medicinal plant, *H. rosa‐sinensis* , to evaluate the cancer‐ameliorating potential. Harini (2024) conducted a trial to investigate the anticancer potential of *H. rosa‐sinensis* by MTT assay, and it was demonstrated that the ethanolic extract of hibiscus (10, 25, 50 μg/mL) showed dose‐dependent attenuation of cancer proliferation and migration. Furthermore, Lu et al. (2022) used *H. rosa‐sinensis* to synthesize silver (Ag) nanoparticles and determined their impact on the viability of hepatic carcinoma. The results revealed that silver nanoparticles had cytotoxic potential against the invasion, migration, and proliferation of hepatocellular cancer cell lines. The IC50 was 223 μg/mL for SNU‐387, 265 μg/mL for Morris hepatoma (McA‐RH7777), 185 μg/mL for hepatic ductal carcinoma (LMH/2A), and 188 μg/mL for Novikoff hepatoma (N1‐S1 Fudr) cell lines.

*H. rosa‐sinensis* depicted cytotoxic activity against human liver cancer (Hep G2) cell lines by revealing suppression of 132.49% (Shafiq et al. 2021). Moreover, Akhtar et al. (2022) prepared gold nanoparticles of hibiscus and curcumin extract and conducted a comparative study against cancerous cells (HCT‐116 and MCF‐7). The results demonstrated the higher cytotoxic activity of hibiscus gold nanoparticles with an IC50 of 5.80 μg/mL and 3.62 μg/mL against the migration and proliferation of HCT‐116 and MCF‐7 cells compared to curcumin gold nanoparticles. It has been observed that mutations in the ESR1 and HER2 genes advance the development of breast cancer in females. Therefore, Agrawal et al. (2021) proposed an *in silico* study to investigate the effect of hibiscus on ESR1 and HER2 in breast cancer management. It has been evident that the bioactive compounds, such as rutin, quercetin, kaempferol, and myricetin, of hibiscus have promising effects on attenuating the impact of ESR1 and HER2; however, among these compounds, rutin has a relatively higher suppressive activity, which acts as an inhibitor of ESR1 and HER2 genes. The prevalence of skin cancer is constantly increasing, which causes 50,000 deaths among individuals all over the world. Melanoma is the principal factor aggravating the pathogenesis of skin cancer. However, effective treatment to manage its etiology is limited. Therefore, alternative and safer techniques for utilizing plants and plant‐based compounds are incorporated into modern trials. Goldberg et al. (2016) investigated the growth inhibitory potential of hibiscus flowers' aqueous extract against B16F10 melanoma cells. The results revealed dose‐dependent suppression of melanoma growth and proliferation by hibiscus supplementation. The findings showed that 1 mg/mL inhibited 2‐fold cell proliferation, while 2 mg/mL reduced 4 times cell growth. Furthermore, Rehana et al. (2017) showed that the copper oxide nanoparticles (CuO NPs) of *H. rosa‐sinensis* had tumor suppressor potential against MCF‐7, Hep‐2, and A549 cancer cell lines. The findings revealed that CuO–S6 showed maximum inhibitory potential with an IC50 of 19.77 μg/mL against MCF‐7 cell lines, whilst, CuO–S3 and CuO–S6 exhibited IC50s of 21.63, 21.66, and 18.11 μg/mL against HeLa, Hep‐2, and A549 cell lines, respectively.

### 9.3 Antidiabetic Potential

Diabetes mellitus, especially type 2 diabetes, is rapidly prevailing and affecting every 1 in 9 individuals globally. According to reports, diabetes affects ~450 million individuals above 30 years old residing in low‐ and middle‐income countries, and these counts are progressively increasing from year to year. According to Yu et al. (2024), diabetes not only negatively affects pancreatic activity but also influences other organs of the body, i.e., eyes (diabetic retinopathy), nerves (diabetic neuropathy), kidneys (diabetic nephropathy), and feet (diabetic foot). Therefore, medicinal plants, such as hibiscus, have been implemented in rodent trials to confirm their antidiabetic potential. The α‐amylase suppressive activity was assessed by a calorimetric method to determine the antidiabetic activity of hibiscus, and it has been proven that the hypoglycemic potential of hibiscus was obtained by inhibiting α‐amylase activity (Harini 2024). The neutralizing effect of *H. rosa‐sinensis* was determined by Sharma et al. (2023) against α‐amylase and α‐glucosidase activity, and it was observed that higher α‐amylase and α‐glucosidase attenuating activity was shown by ethyl acetate extract with IC50 values of 83 μg/mL and 53.33 μg/mL. Previously, Ansari et al. (2020) used the ethanolic extract of *H. rosa‐sinensis* to investigate the hypoglycemic effect in type 2 diabetic rats. The attenuation in blood glucose, triglycerides, and cholesterol has been observed, along with improved HDL cholesterol and hepatic glycogen, by administering 250/500 mg/kg body weight *of H. rosa‐sinensis*. Additionally, *H. rosa‐sinensis* inhibited the HPP‐IV enzyme to significantly improve glucose tolerance by lowering glucose absorption and postprandial hyperglycemia. Studies evidencing the antidiabetic potential of *H. rosa‐sinensis* are mentioned in Table 2.

**TABLE 2:** Antidiabetic and hypoglycemic potential of *H. rosa‐sinensis* .

Study	Part used	Extract	Subjects	Dose	Upregulation	Downregulation	References
In vitro	Petals		RIN‐m5F pancreatic β‐cells	—	Insulin excretion, Ucn‐3, Pdx‐1, MafA, foxO‐1, and Nkx6.1 expression	NF‐κB translocation	Pillai and Mini (2018)
In vitro	Flowers	Ethyl acetate	3 T3‐L1 cells	25 & 50 μg/mL	AMPK, β‐oxidation of fatty acids	Triglycerides, PPAR‐γ, C/EBPα, SREBP‐1c, acetyl‐CoA carboxylase	Lingesh et al. (2019)
In vivo	Flowers	Aqueous	Female Wistar rats	300 mg/kg body weight (BW)/day	Maternal and fetal weight	atherogenic index, coronary artery risk index	Afiune et al. (2017)
In vivo	Flower	Ethanol	Streptozotocin induced rats	250 mg/kg BW	HDL‐cholesterol, insulin release	Blood glucose and insulin levels, cholesterol, triglycerides	Sachdewa and Khemani (2003)
In vivo	flowers	Hydroalcoholic	Alloxan‐induced diabetic Wister rats	50, 100, and 200 mg/kg BW	Size, necrosis and atrophy of Islets cell	Total cholesterol and triglycerides	Pethe, Yelwatkar, Gujar, et al. (2017)
In vivo	leaves	Aqueous methanol	Streptozotocin‐induced diabetic rats	400 mg/kg BW	HDL‐cholesterol, insulin release	LDL‐cholesterol, total cholesterol, triglycerides	Zaki et al. (2017)
In vivo	leaves	Ethanol	Alloxan‐infused diabetic rats	0.5, 1, and 2 mg/kg BW	HDL‐cholesterol	Glucose levels, total cholesterol, triglycerides, LDL‐cholesterol	Mamun et al. (2013)
In vivo	Roots		alloxan induced diabetic rats	500 μg	Insulin secretion	Blood glucose and lipids concentrations, triglycerides	Kumar et al. (2013)
In vivo	flowers	Ethanol	alloxan induced diabetic rabbits	50, 100, and 200 mg/kg BW	Dyslipidemia	Lipids particularly total cholesterol and triglycerides	Pethe, Yelwatkar, Manchalwar, and Gujar (2017)
In vivo	flowers	Aqueous	Streptozotocin induced diabetic rats	250–500 mg/kg BW	Hypoglycemia	Serum glucose, glycosylated hemoglobin, lipids levels	Bhaskar and Vidhya (2012)
In vivo	leaves	Aqueous	Streptozotocin induced diabetic rats	250 mg per kg BW	Glucose tolerance, hypoglycemia	Blood glucose levels	Sachdewa et al. (2001)
In vivo	flowers	Aqueous	Pregnant diabetic rats	100, 200, and 400 mg/kg BW	Hypolipidemia and hypo‐insulinemia	HDL‐cholesterol, ALT and triglycerides	Silva et al. (2015)
In vivo	flowers	Ethanol	Streptozotocin‐induced Wister albino rats	125 mg/kg BW	Necrosis and degeneration of pancreatic β‐cells	Blood sugar and lipids	Chauhan and Rani (2024a)
In vitro and In silico	Flower	Ethyl acetate	—	—	Hypolipidemic effect	α‐glucosidase and α‐ainilase enzymes	Sharma et al. (2023)
In vivo	Petals	Acidified methanol	Streptozotocin induced diabetes in male Sprague–Dawley rats	50 mg/kg BW	Antioxidant enzymes (superoxide dismutase, catalase, glutathione peroxidase, and glutathione)	Glucose, glycated hemoglobin, lipids, oxidative stress	Kalpana et al. (2021)
In vivo	Flowers	Aqueous	Streptozotocin‐triggered diabetic rats	50, 100, 200, and 1000 mg/kg BW	Body weight	IL‐6, IL‐1β, TNF‐α, blood sugar levels	Oluwamodupe et al. (2024)

### 9.4 Anti‐Inflammatory and Cardioprotective Properties

Inflammation is the response of immune cells in case any foreign pathogen enters the body. T‐cells play a beneficial role in killing these pathogens, which are involved in the progression of certain inflammatory disorders, i.e., inflammatory bowel disease, multiple sclerosis, diabetes, cancer, autoimmune disorders, cardiovascular disorders, and rheumatoid arthritis. The CD4^+^ and CD8^+^ cells differentiate self or foreign proteins, which helps T cells advance their activity (Chen et al. 2024; Mahdy et al. 2024). The comparative study investigated the anti‐inflammatory potential of ethanolic extract of *H. rosa‐sinensis var alba* (white Hibiscus) and *H. rosa‐sinensis L*. flowers and leaves by supplementing 5, 50, 100, and 1000 mg/kg extract in carrageenan‐induced paw edema. The results showed significant alleviation of inflammatory markers, particularly cyclooxygenase levels (Guddeti et al. 2015; Raduan et al. 2013).

In another study, Singh et al. (2018) revealed that the tea made from *H. rosa‐sinensis* has inflammation‐alleviating properties, reducing cartilage destruction and preventing inflammation‐induced arthritis. Previously, Kandhare et al. (2012) investigated the ameliorating potential of *Hibiscus* leaves' hydroalcoholic extract in acetic acid‐stimulated colitis in male Wistar rats by administering 50, 100, and 200 mg/kg *of H. rosa‐sinensis*. It has been observed that oxidative stress, MDA, MPO, NO, and TNF‐α reduced while SOD and GSH levels improved by 100 and 200 mg/kg doses of Hibiscus. Similarly, Sruthi et al. (2021) conducted an in vitro study to determine the anti‐inflammatory and anti‐arthritic potential of *H. rosa‐sinensis* leaves' ethanolic extract. The results depicted that *H. rosa‐sinensis* stabilizes the membrane (94%) and suppresses protein denaturation (89%) within the specific dose of 500 μg/mL, thereby showing effectiveness against inflammation and arthritis. Moreover, the ethanolic extract of *H. rosa‐sinensis* roots was employed to investigate the analgesic and anti‐inflammatory potential in carrageenan‐induced paw edema by supplementing 250 and 500 mg/kg root extract and concluded that pain and inflammation were significantly attenuated as the concentration of the extract was increased, revealing a dose‐dependent effect (Begum et al. 2018).

Additionally, Gupta et al. (2014) depicted that the oral supplementation of ~250 mg/kg aqueous root extract of *H. rosa‐sinensis* ameliorated inflammation in carrageenan‐induced paw edema in Swiss albino rats in a dose‐dependent manner. Gulati (2022) assessed the anti‐inflammatory mechanism of *H. rosa‐sinensis* and *Piper nigrum* using a bronchial asthma model and by supplementing 100 and 250 mg/kg *H. rosa‐sinensis* and 30 and 100 mg/kg *Piper nigrum* for 2 weeks. Significant reductions in IgE, TNF‐α, and *P‐enh* levels were observed, and these plants also ameliorated all the blood and bronchoalveolar lavage fluid parameters, ultimately relieving inflammation and bronchospasm in bronchial asthma. The adipogenic potential of *H. rosa‐sinensis* ethyl acetate flower extract was investigated in 3 T3‐L1 cells by Lingesh et al. (2019), who administered 25 and 50 μg/mL and found the modulated AMP‐activated protein kinase (AMPK), and reduced expression of triglycerides, lipolysis, PPAR‐γ, Sterol regulatory element‐binding protein‐1c (SREBP‐1c), Acetyl‐CoA carboxylase, and fatty acid synthase. Later, Jeevan Kumar and Rajeshkumar (2022) reported the anti‐inflammatory property of silver nanoparticles synthesized from *H. rosa‐sinensis* , which was 79% at the concentration of 50 μL. A previous study conducted by Somchit et al. (2008) showed that the leaves of *H. rosa‐sinensis* have dose‐dependent amelioration of prostaglandin D2‐induced inflammation from the paw.

In addition, Gauthaman et al. (2006) conducted a study to assess the potential of 2% CMC‐suspended *H. rosa‐sinensis* flower against inflammation and oxidative stress‐induced myocardial ischemic reperfusion injury in Wister albino rats of 150–200 g by supplementing hibiscus suspension at the specific dosage of 125, 250, and 500 mg/kg for 6 days a week for 1 month. It has been evident that thiobarbituric acid reactive substances and SOD are significantly improved in the 250 mg/kg administered group, while GSH and CAT levels are relatively alleviated. However, no significant influence was observed in the 125 and 500 mg/kg treated groups.

Previously, Khandelwal et al. (2011) investigated the effect of *Hemidesmus indicus* and *H. rosa‐sinensis* at 90, 180, and 360 μg/mL concentrations against ischemia–reperfusion injured rats. Short‐term improvements in left ventricular developed pressure and coronary flow were observed by *Hemidesmus indicus*. However, *H. rosa‐sinensis* significantly enhances left ventricular developed pressure and coronary flow, having more vasodilatory, inotropic, and cardioprotective potency than *Hemidesmus indicus*. In another trial, the impact of anthocyanins from *H. rosa‐sinensis* against hypertension and deoxycorticosterone acetate‐salt‐induced oxidative stress was evaluated in rats by supplementing 100 and 300 mg/kg hibiscus for a month. The results revealed that the supplementation significantly ameliorated systolic blood pressure, oxidative stress, and vascular reactivity changes (Mohan et al. 2011).

**FIGURE 3:**
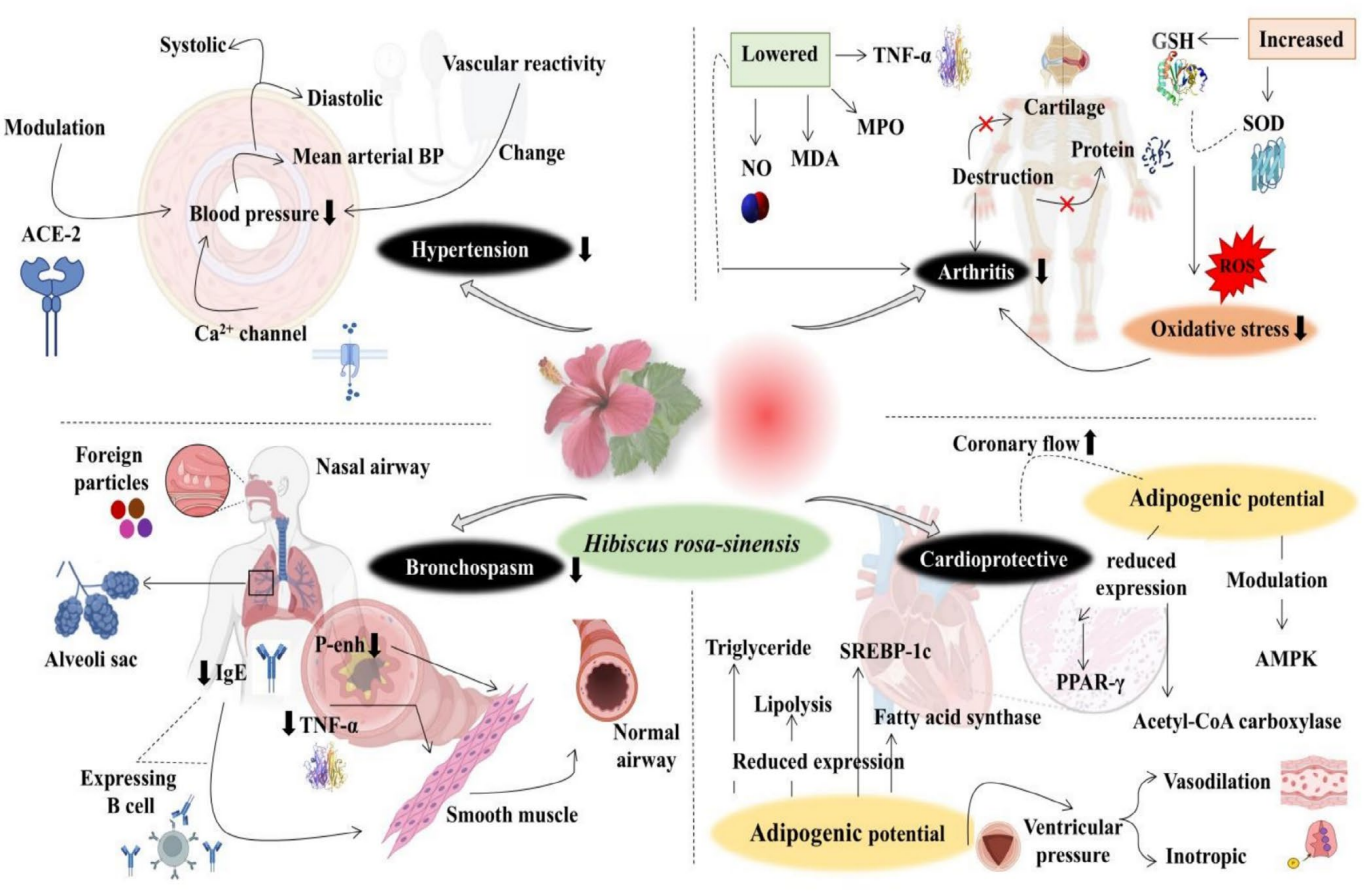
Anti‐inflammatory and Cardioprotective potential of *Hibiscus rosa‐sinensis* via modulating hypertension, suppressing pro‐inflammatory markers and oxidative stress, inhibiting IgE, improving antioxidant enzymes, and reducing PPAR‐ γ.

Furthermore, Amtaghri et al. (2022) investigated the hypotensive and vasodilatory potential of *H. rosa‐sinensis* in hypertensive rats by providing 100 mg/kg aqueous flower extract of *H. rosa‐sinensis* for 7 days. The results showed that oral consumption of hibiscus flower extract attenuated arterial blood pressure. Moreover, the modulation of angiotensin‐converting enzyme‐2 and the Ca^2+^ channel suppression pathway promoted vasodilation and ultimately reduced hypertension. The anti‐inflammatory and cardioprotective role of *H. rosa‐sinensis* is illustrated in Figure 3.

### 9.5 Antimicrobial Properties

Microorganisms are found all over the world with their distinct and versatile nature. Some of them, such as *Bifidobacterium* and *Lactobacillus*, have beneficial effects and play their role in improving the health of individuals. Fermentation, the process to enhance and improve the bioavailability of nutrients, is driven by the beneficial microbial population. However, *Escherichia coli* and *Staphylococcus aureus* are harmful and pathogenic, causing numerous health disorders. Various antimicrobial drugs are commercially employed to reduce their severity. The antimicrobial attributes of *H. rosa‐sinensis* have been reported in multiple studies. Chai (2020) evaluated the antibacterial potential of orange, white, and hybrid hibiscus leaves and flowers against *Staphylococcus aureus* and *Escherichia coli* . It was observed that the largest zone of inhibition was shown by the hybrid flower (18.67 mm) and the orange flower (16 mm) for the respective bacterial strains, i.e., *Staphylococcus aureus* and *Escherichia coli* . In another study, Abate and Belay (2022) investigated the antimicrobial activity of *H. rosa‐sinensis* against *E. coli* , *S. aureus* , *K. pneumoniae* , and *S. epidermidis* . The growth inhibitory effect was recorded to be 6.33–11.50 mm for *S. aureus* and *S. epidermidis* , while it was 6.67–13 mm against *E. coli* and *K. pneumoniae* . Similarly, Ruban and Gajalakshmi (2012) conducted an in vitro study to inquire about the antimicrobial potential of *H. rosa‐sinensis* flower extract by disc and agar diffusion methods. It has been found that the growth of *Bacillus subtilis* and *Escherichia coli* was alleviated by cold extraction of hibiscus with an inhibition zone of 17 and 14.50 mm, respectively. Additionally, the methanol extract of hibiscus showed the maximum bactericidal effect against *B. subtilis* and *E. coli* , having a zone of inhibition of 18.86 and 18 mm, respectively. However, the ethanol extract showed the maximum zone of inhibition at 20.40 mm against *Salmonella* species. Furthermore, using the agar disc diffusion method, Mak et al. (2013) investigated the antibacterial attributes of *H. rosa‐sinensis* and *Senna bicapsularis* flower extracts. *H. rosa‐sinensis* ameliorated the growth of food‐borne pathogens, including *Salmonella typhimurium* and *Staphylococcus aureus* , while *Senna bicapsularis* suppressed the growth of *Bacillus cereus* and *Klebsiella pneumoniae* . Further studies regarding the antimicrobial agent of *H. rosa‐sinensis* are presented in Table 3.

**TABLE 3:** Antimicrobial properties of *H. rosa‐sinensis* .

Part	Extract/particles	Method	Pathogen/Microorganism	Zone of inhibition	References
Flowers	Petroleum ether	Disc diffusion	methicillin‐resistant *Staphylococcus aureus*	18.6 mm	Arullappan et al. (2009)
Flowers	Ethanol and methanol	Agar disc diffusion	*Staphylococcus* sp., *Bacillus* sp., and *Escherichia coli*	12.75 mm to 16.75 mm	Khan et al. (2014)
Leaves	Solvent	Agar disc diffusion	*Bacillus subtilis* and *Staphylococcus aureus*	18.82 mm and 11 mm	Udo et al. (2016)
Leaves and flowers	Ethanol	Agar well diffusion and bacteriological enumeration	*S. aureus* and *Salmonella typhimurium*	Flowers have more inhibition zone than leaves	Uddin et al. (2010)
Flowers	Ethanol & ethyl acetate	24‐well plates with Brucella agar medium	*Helicobacter pylori*	MIC = 0.2–0.25 mg/mL	Ngan et al. (2021)
Flowers	Iron oxide nanoparticles	Agar well diffusion	*Staphylococcus aureus* , *Pseudomonas aeruginosa* , *Klebsiella pneumonia*, and *Escherichia coli*	2 mm to 6 mm	Buarki et al. (2022)
Leaves	Zinc oxide and titanium dioxide nanoparticles	Disc diffusion	*E. coli* and *S. aureus*	82.3 mm for *E. coli* and 54.3 mm for *S. aureus*	Abd El‐Kader et al. (2021)
Leaves and flowers	Ethanol	Agar disc diffusion	*Staphylococcus epidermidis* , *Staphylococcus aureus* , and *Staphylococcus epidermidis*	MIC = 20 mm for *Staphylococcus epidermidis* , and *Staphylococcus aureus* , while Minimum Bactericidal Concentration = 20 mg/mL for *Staphylococcus epidermidis*	Seyyednejad et al. (2010)
leaves	CoFe_2_O_4_ nanoparticles	Disc diffusion method	*S. aureus‐9779* and *E. coli‐745*	8 mm and 12 mm for respective *S. aureus‐9779* and *E. coli‐745*	Velayutham et al. (2022)
Leaves	Silver (Ag) and gold (Au) nanoparticles	Agar well diffusion	*Pseudomonas aeruginosa* , *Bacillus subtilis Micrococcus luteus* , *Staphylococcus epidermidis* , *Staphylococcus aureus* , *Enterobacter aerogenes* , *Escherichia coli* , *Streptococcus pneumoniae* , and *Aeromonas hydrophilia*	Inhibition zone (0.04 cm to 0.41 cm) by silver nanoparticles and 0.09 cm to 0.23 cm by gold nanoparticles	Tyagi et al. (2017)
Flowers	Aqueous	Agar well diffusion	*P. aeruginosa* , *Serratia*, *Micrococcus*, *Enterobacter*, and *Salmonella*	Inhibition zone ranges from 33 mm to 62 mm	Al‐Alak et al. (2015)
Leaves	Zinc oxide nanoparticles	Agar well diffusion	*Escherichia coli* and *Staphylococcus aureus*	Growth inhibition zone is 35 mm for *Escherichia coli* and 20 mm for *Staphylococcus aureus*	Elemike et al. (2021)
Leaves	NiSe nanoparticles	Well diffusion	*Escherichia coli* and *Staphylococcus aureus*	10 and 15 mm for the respective *Escherichia coli* and *Staphylococcus aureus*	Velayutham et al. (2023)
Flowers	Ethanol	Well diffusion	*Staphylococcus aureus MTCC 87*, *Bacillus cereus MTCC 430*, *Clostridium perfringens MTCC 450*, *Listeria monocytogenes MTCC 657*, *Escherichia coli MTCC 43*, *Salmonella typhi MTCC 1264*, and *Pseudomonas aeruginosa MTCC424*	16 mm, 13 mm, 10 mm, 14 mm, 17 mm, 12 mm, and 11 mm for *Staphylococcus aureus MTCC 87*, *Bacillus cereus MTCC 430*, *Clostridium perfringens MTCC 450*, *Listeria monocytogenes MTCC 657*, *Escherichia coli MTCC 43*, *Salmonella typhi MTCC 1264*, and *Pseudomonas aeruginosa MTCC424* respectively	Karnwal (2022)
Flowers	Silver nanoparticles	Agar well diffusion	*A. hydrophila*	16 mm	Surya et al. (2016)
Leaves	Aqueous	Well diffusion	*Streptococcus pyogenes* and *Klebsiella pneumoniae*	1.5 and 1.7 cm for the respective *Klebsiella pneumoniae* and *Streptococcus pyogenes*	Vignesh and Nair (2018)
Flowers	Co_3_o_4_ nanoparticles	Diffusion	*Staphylococcus aureus* , *Streptococcus mutans* , *Klebsilla pneumonia*, *E. coli* , *Aspergillus flavus*, and *Aspergillus niger*	—	Anuradha and Raji (2019)
Leaves	Ethyl acetate	Agar diffusion	*Staphylococcus aureus* , *Bacillus subtilis* , *Streptomyces alboniger* , *Micrococcus luteus* , *Staphylococcus epidermis*, *Pseudomonas aeruginosa* , and *Bordetella bronchiseptica*	Orange cultivars revealed MIC values for *Staphylococcus aureus* (20 mg/mL), *Bacillus subtilis* (2.5 mg/mL), *Streptomyces alboniger* (10 mg/mL), *Micrococcus luteus* (2.5 mg/mL), *Staphylococcus epidermis* (2.5 mg/mL), *Pseudomonas aeruginosa* (1.25 mg/mL), and *Bordetella bronchiseptica* (1.25 mg/mL)	Nagar (2012)
Petals	Silver nanoparticles	Disc diffusion	*Escherichia coli* , *Staphylococcus aureus* , and *Klebsiella pneumonia*	*E. coli* (98%), *S. aureus* (30%), and *K. pneumonia* (37%)	Nayak et al. (2015)
Flowers	Copper oxide nanoparticles	Well diffusion	*Klebsiella pneumoniae* , *E. coli* , *Shigella flexneri* , and *Bacillus subtilis*	—	Rajendran et al. (2018)
Flowers	Methanol and ethanol	Disc diffusion	*Staphylococcus aureus* and *Escherichia coli*	Hybrid flowers showed maximum zone of inhibition (18.67 mm) for *Staphylococcus aureus* while orange flowers revealed maximum inhibition zone (16 mm) for *Escherichia coli*	Tong and Tee (2022)
Leaves	Ethanol	Agar well diffusion	*Streptococcus mutans* and *Lactobacillus acidophilus*	6.25 μg/mL and 25 μg/mL for *S. mutans* and *L. acidophilus*	Nagarajappa et al. (2015)
Leaves, flowers, and roots	Methanol and aqueous	Agar well diffusion	*Streptococcus aureus*	Methanolic leaves extract revealed highest zone of inhibition (29 mm), subsequently methanol flower extract (17 mm), aqueous flower extract (14 mm), and methanolic root extract (13 mm)	Priya and Sharma (2021)
Leaves	Aqueous	Agar well diffusion	*Escherichia coli* , *Salmonella typhimurium* , *Bacillus subtilis* , and *S. aureus*	14.5 mm, 14 mm, 11.50 mm, and 16 mm for *E. coli* , *S. typhimurium* , *B. subtilis* , and *S. aureus*	Dowara et al. (2024)
Leaves essential oil	essential oil	Disc plate and agar well diffusion	*Klebsiella* sp., *Pseudomonas aeruginosa* , and *Fusarium oxysporum*	18.5, 11.5, and 23 μg/mL for *Klebsiella* sp., *Pseudomonas aeruginosa* , and *Fusarium oxysporum*	Sidhu et al. (2023)
Leaves	Silver nanoparticles	Agar well diffusion	*Aeromonas hydrophilia*	11 mm	Vijayaraj and Kumaran (2017)
Leaves	Ethanol	Agar well diffusion	*E. coli* and *Staphylococcus aureus*	2.5 and 5 mg for *E. coli* and *S. aureus*	Ghadigaonkar (2023)
Flowers	Aqueous	Well diffusion	*Streptococcus sanguinis*	6.35 mm	Farasayu et al. (2021)
Leaves	Water	Disc diffusion and micro‐dilution	*Limosilactobacillus fermentum MA‐7*	6.85 mm to 10.74 mm	Sağlam et al. (2023)
Stem bark	Methanol	Agar well diffusion	*Staphylococcus aureus* and *Escherichia coli*	50 and 200 mg/mL for *S. aureus* and *E. coli*	Umar et al. (2024)
Flower	Water and methanol	Agar disc diffusion	*Bacillus cereus* , *Staphylococcus aureus* , *Salmonella typhimurium* , *E. coli* , and *K. pneumonia*	11 mm, 15 mm, 9 mm, 14 mm, and 12.5 for the respective microbes	Rassem et al. (2024)
Seeds	Oil	Agar well diffusion	*K. pneumonia*	6 mm at 10 μg/mL, 16 mm at 25 μg/mL, and 25 mm at 50 μg/mL	Yang et al. (2020)
Leaves	CoFe_2_O_4_ nanoparticles	Disc diffusion	*S. aureus* , *B. Subtillis*, *and E. coli*	15, 20, and 7 μg/mL at 100 mm concentration for the respective strains	Singaravelan et al. (2024)
Leaves	Ethanol	Disc diffusion	*Candida albicans* , *Candida tropicalis* , and *Candida glabrata*	9 mm, 8 mm, and 9 mm respectively	Zuhaira et al. (2020)
Flowers	Chloroform	Agar well diffusion	*Candida albicans*	26.6 mm	Mohana et al. (2024)
Flowers	Aqueous	Diffusion	*Staphylococcus aureus* , *Bacillus Subtilis* , *Pseudomonas Aeruginosa* , *and Escherichia coli*	17, 17, 16, and 18 mm for the selected strains at 100 μg/mL	Magalakshmi et al. (2022)
Flowers	Ethanol	Disc diffusion	*Taphylococcus epidermidis* and *Staphylococcus saprophyticus*	—	Patrice et al. (2017)

### 9.6 Hepatoprotective Potential

The liver performs various functions, including detoxification of toxins, drugs, and other deleterious metabolites; metabolism of nutrients; digestion and absorption by producing bile; storage of glucose, vitamins, and minerals; and hormone regulation in the body to modulate the homeostatic environment (Rakhi et al. 2022). Medicinal plants such as *H. rosa‐sinensis* are investigated for improving liver health, whose evidence is described comprehensively here. Kumar (2020) examined the hepatoprotective effect of ethanolic and aqueous flower extract of *H. rosa‐sinensis* in carbon tetrachloride (CCl4) induced liver injury in rats. It has been shown that alanine transaminase (ALT), alkaline phosphatase (ALP), aspartate transaminase (AST), triglycerides (TG), bilirubin, and cholesterol levels were significantly alleviated by the administration of ethanol (200 mg/kg) and aqueous (400 mg/kg) flower extract of hibiscus. The comparative study determined the role of aqueous leaf extract of *H. rosa‐sinensis* and aqueous peel extract of pomegranates against liver disorders and CCl4‐induced oxidative stress in male albino rats. It has been shown that the supplementation of aqueous extract of *H. rosa‐sinensis* leaf (250, 500, and 750 mg/kg body weight) and pomegranate peel (100, 200, and 300 mg/kg body weight) has a similar influence in the alleviation of liver functional parameters. ALT levels were reduced from 29.55 to 20.15 U/L, AST from 47.97 to 30.99 U/L, ALP from 305.96 to 170.55 U/L, and total bilirubin from 3.11 mg/dL to 2.21 mg/dL (El‐Sayed 2018).

Similarly, Sattar Ali (2022) evaluated the hepatic protective effect of *H. rosa‐sinensis* zinc oxide nanoparticles and hibiscus extract on liver tissue and DNA fragmentation in adult male Wister rats (*n* = 35). The results showed that the subcutaneous administration of zinc oxide nanoparticles (75 and 100 mg/kg body weight) has comparatively increased AST, ALP, and ALT activity compared to zinc oxide nanoparticles at a 25 mg/kg body weight concentration. However, hibiscus extract has a limited attenuating impact on AST, ALP, and ALT. Previously, Biswas et al. (2014) explored the hypocholesterolemic potential of aqueous flower extract of *H. rosa‐sinensis* in diet‐induced hypercholesterolemia in 180–230 g male Wister rats (*n* = 42). It has been observed that body weight, AST, ALT, ALP, total protein, and MDA levels were reduced by consuming 240 mg/kg of body weight per day for a month. Lu et al. (2022) also synthesized silver nanoparticles from *H. rosa‐sinensis* and checked their impact on managing hepatic carcinoma. Silver nanoparticles showed dose‐dependent cytotoxic activity against hepatocellular cancer (SNU‐387), Morris hepatoma (McA‐RH7777), hepatic ductal carcinoma (LMH/2A), and Novikoff hepatoma (N1‐S1 Fudr).

Furthermore, Nwibo et al. (2016) evaluated the potential of *H. rosa‐sinensis* leaf extract on hyperlipidemic rats' liver and blood indices. They concluded that the leaf extract had not influenced the hematological parameters (hemoglobin, packed cell volume, and red blood counts). However, total cholesterol and LDL levels were significantly attenuated, and the hibiscus leaf extract improved HDL levels. In another study, Sahu (2016) determined the protective effect of *H. rosa‐sinensis* alcoholic leaf extract against piroxicam‐induced liver toxicity in adult Swiss albino mice (*n* = 60) by administering 30 mg/kg alcoholic leaf extract for 2 weeks. The outcomes demonstrated that the leaf extract of *H. rosa‐sinensis* decreased elevated ALT, AST, ALP, and hepatic lipid peroxidation levels. Similarly, Hussain et al. (2017) revealed that the cadmium‐induced hepatic toxicity in adult male albino rabbits was significantly ameliorated by the flavonoid‐enriched *H. rosa‐sinensis* leaves and flowers (200 mg/kg/day for 2 months), thereby demonstrating the hepatoprotective potential. Previously, Gomathi et al. (2008) exposed that the flower petals of *H. rosa‐sinensis* lowered the levels of monosodium glutamate, which triggered elevation of free fatty acids, TG, TC (total cholesterol), LDL, and VLDL (very low‐density lipoprotein). Similar results were observed in triton and atherogenic diet‐induced hyperlipidemic rats by the oral supplementation of 500 mg/kg body weight ethanolic flower extract of *H. rosa‐sinensis* (Sikarwar Mukesh and Patil 2011).

**FIGURE 4:**
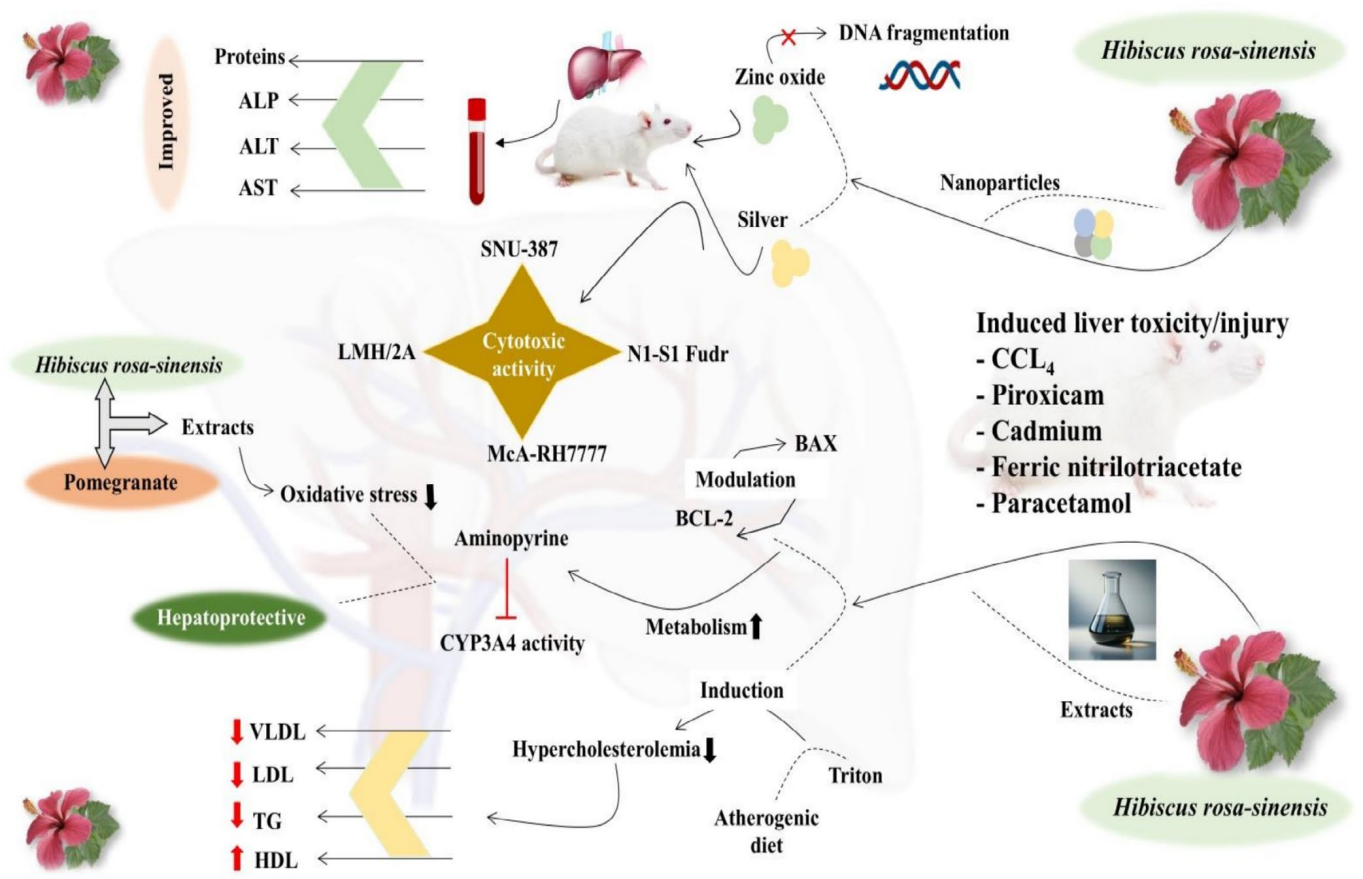
Hepatoprotective mechanism of *Hibiscus rosa‐sinensis* via reducing liver enzymes, alleviating oxidative stress, modulating CYP3A4 activity, regulating BCL‐2 and BAX expression, and lowering LDL, VLDL, TGs.

Recently, Dayal et al. (2022) used an aqueous extract of *H. rosa‐sinensis* and *Butea monosperma* in their hepatoprotective investigation against ferric nitrilotriacetate‐induced toxicity in rats. Liver functional biomarkers (ALT, AST, and ALP), triglycerides, lipids, proteins, and oxidative markers were alleviated by both extracts. In another investigation, Han et al. (2020) described that the methanolic extract of *H. rosa‐sinensis* improved aminopyrine metabolism by elevating hepatic CYP3A4 activity. In a similar year, Adil and Manampiring (2020) inquired about the potential of 1 mL polar (0.075 g mg/200 g BB/day in 0.5% CMC suspension) and non‐polar flowers extract of hibiscus (0.075 mg/200 g BB/day in 1% CMC suspension) on paracetamol‐stimulated hepatic toxicity in male white rats for 8 days. It has been observed that polar and non‐polar hibiscus extracts maintain the levels of SGOT and SGPT. Similarly, Rajavelu and Bs (2024) showed that the ethanolic leaf extract of *H. rosa‐sinensis* ameliorated liver cancer by inducing apoptosis through modulating BAX and BCL‐2. The hepatoprotective activity of *H. rosa‐sinensis* is shown in Figure 4.

### 9.7 Antidepressants and Neuroprotective Activity

Anxiety and neurodegenerative disorders are reducing the healthy and normal life expectancy of individuals. Oxidative stress and inflammation are provoked by several factors, such as processed and fried food consumption, prolonged and repeated infections, aging, neuroinflammation, and heavy metals exposure, which induce neurodegenerative disorders (Castillo‐Rangel et al. 2023; Angelopoulou et al. 2022; Dolgacheva et al. 2022). The effect of *H. rosa‐sinensis* in mitigating neurological symptoms and its associated disorders has been reported. Shewale et al. (2012) proposed a study to evaluate the antidepressant capability of methanolic flower extract of flavonoid (anthocyanins) enriched *H. rosa‐sinensis* (30 and 100 mg/kg) by tail suspension and forced swim test. It has been observed that the duration of Haloperidol, Prazosin, and *p‐chlorophenyl alanine‐*stimulated immobility was decreased in both tests, confirming antidepressant activity. Similarly, Khalid et al. (2014) inquired about the antidepressant activity of ethanol flower extract of *H. rosa‐sinensis* at 100, 250, and 500 mg/kg doses by conducting forced‐induced swimming, tail suspension, and open field tests. The results revealed that all the supplemented dosages reduced immobility duration in forced‐induced swimming and tail suspension tests, while no significant effect was observed in open field tests.

Moreover, Sheikhar et al. (2024) assessed the antidepressant potential of *H. rosa‐sinensis* leaves in Swiss albino mice of 20 to 35 g by administering 200 and 400 mg/kg extract for 2 weeks and concluded that the ethanolic extract (400 mg/kg) of *H. rosa‐sinensis* leaves has antidepressant potential. Sucharitha and Nagamani (2021) conducted a forced swimming test, tail suspension test, and sleep‐induced test to determine the anxiolytic role of ethyl acetate flower and hibiscus leaf extract. The immobility time was attenuated dose‐dependently in all the selected tests with the oral intake of 100 and 200 mg/kg extract dose, demonstrating anxiolytic effects through adrenergic, dopaminergic, and serotonergic mechanisms. The antidepressant effect of ethanolic hibiscus extract (500 mg/kg) was revealed in mice by suppressing ionotropic GABA receptors (Begum and Younus 2018). In another study, Vijayanand et al. (2018) prepared solid lipid nanoparticles from *H. rosa‐sinensis* . They evaluated their antidepressant activity in male Swiss albino mice and found that solid lipid nanoparticles ameliorated immobility time more than their crude extract.

The neuroprotective capability of methanolic extract of *H. rosa‐sinensis* against the bilateral common carotid artery (BCCA) occlusion model of global cerebral ischemic‐reperfusion was evaluated by supplementing 100, 200, and 300 mg/kg/day for six consecutive days. The outcomes revealed that SOD, CAT, and GSH activity was reduced by hibiscus extract, ultimately reducing anxiety as well as modulating learning and memory (Nade et al. 2010). Previously, Nade et al. (2009) demonstrated that the methanolic roots extract of *H. rosa‐sinensis* (100 to 300 mg/kg) lowered lipid peroxidation and upregulated SOD, CAT, and GSH levels in reserpine‐induced neurobehavioral modifications in the brain. Recently, Shen et al. (2021) showed that the Quercetin 3‐O‐sophoroside isolated from *H. rosa‐sinensis* improved learning and memory in Alzheimer‐affected mice. Moreover, neuronal impairment in the hippocampal CA1 region and SCOP‐induced reduction in ChAT and ACh expression and AChE expression were improved and reversed by Quercetin 3‐O‐sophoroside.

## 10 Miscellaneous Properties

*H. rosa‐sinensis* has several other health‐promoting properties besides its antioxidant, antimutagenic, anti‐inflammatory, antimicrobial, cardioprotective, and neuroprotective characteristics. Soni and Gupta (2011) assessed the aqueous root extract of *H. rosa‐sinensis* as an antipyretic agent against yeast‐provoked pyrexia in Swiss albino rats using the flicking method. The results showed that the intake of 250 mg/kg body weight aqueous root extract has a dose‐dependent effect in attenuating pyrexia. Previously, Sawarkar et al. (2009) revealed the temperature‐lowering potential of aqueous (100 mg/kg) and alcoholic (200 mg/kg) extract of *H. rosa‐sinensis* in pyretic Wister rats. Moreover, Daud et al. (2016) demonstrated the antipyretic property of aqueous extract (500 mg/kg body weight) of *H. rosa‐sinensis* . Similarly, Aziz et al. (2021) inquired about the underlying temperature ameliorating potential of the ethanolic extract of white and red colored *H. rosa‐sinensis* . It has been noticed that the administration of 5 and 50 mg/kg white flower extract significantly alleviated the total temperature in rats.

Vasudeva and Sharma (2008) described that the ethanolic root extract of *H. rosa‐sinensis* at the concentration of 400 mg/kg body weight has potent anti‐implantation and uterotropic activity. Similarly, Jana et al. (2013) demonstrated that the crude flower extract of *H. rosa‐sinensis* at 300 mg/kg for 30, 45, and 60 days resulted in the deteriorating germinal epithelium of testes, consequently revealing antifertility in male albino rats (*n* = 84). Additionally, Kareem et al. (2022) and AL‐Azawi and Al‐hady (2020) demonstrated that the nanoparticles of *H. rosa‐sinensis* and the flower extract of *H. rosa‐sinensis* reduced fertility in male albino rats. Furthermore, Gupta and Yadav et al. (2024) investigated the potential of oral supplementation of *H. rosa‐sinensis* aqueous, ethanol, and benzene leaf extract (100 mg/kg body weight per day for 35 days) on the reproductive health of male albino rats. It has been shown that the benzene extract modified the testis, seminal vesicle, and epididymis and ameliorated spermatogenesis and fertility.

The petroleum ether leaves and flower extract of *H. rosa‐sinensis* were employed by Rose et al. (2020) to evaluate their effect on the hair growth of Dawley Sprague rats for 7 weeks. It has been noticed that the leaf extract has comparatively excessive hair growth compared to the flower extract of *H. rosa‐sinensis* . Later, Lailiyah (2023) used ethanol leaf extract of *H. rosa‐sinensis* in his study to prepare the cream for hair growth and investigated its impact on the hair growth of white rabbits and concluded that the cream at 20% concentration produced more hairs than at other concentrations, i.e., 10% and 15%. In a recent investigation, Khadasare et al. (2024) formulated a serum by combining hibiscus flowers and leaves, olive oil, peppermint oil, amla powder, vitamin E, curry leaves, lavender oil, and coconut oil, which promoted hair growth, hair quality, and strengthened them. Similarly, Parihar et al. (2024) revealed that the formulations made by *H. rosa‐sinensis* flowers, *Allium cepa* bulbs, *Eclipta alba* leaves, and *Trigonella foenum‐graecum* seeds improved hair growth as well as ameliorated hair fall.

## 11 Synergistic Role With Other Plants

The potency and effectiveness of plants and compounds are significant challenges directly or indirectly influenced by various factors, such as genetics, bioavailability, administration route, dose, and formulations. Recent debates have continued developing novel alternative strategies for improving the efficiency of plants. Multiple studies have been conducted on evaluating the synergetic significance of plants and compounds, and it has been observed that the plants/compounds supplemented in combination have comparatively better outcomes than plants/compounds supplemented alone. For instance, the antimicrobial potential of *H. rosa sinensis* leaves oil and its metabolite, dioctyl phthalate, was evaluated synergistically against *Klebsiella* species, *Pseudomonas aeruginosa* , and *Fusarium oxysporum* using disc plate and agar well diffusion method. The results revealed higher antimicrobial activity by exhibiting MICs of 18.5, 11.5, and 23 μg/mL against the respective bacterial and fungal strains (Sidhu et al. 2023).

Moreover, diabetes mellitus is prevailing throughout the world, which affects other organs of the body, particularly reproductive health among males. Chauhan and Rani (2024b) investigated the combination effect of *H. rosa sinensis* and camel milk on reproductive health in diabetic Wistar rats for 4 weeks. The results showed that pancreatic and seminiferous tubule damage caused by diabetes was significantly alleviated by the synergistic provision of camel milk and *H. rosa sinensis*. Furthermore, the supplementation has improved sperm motility, count, and viability, thereby modulating the reproductive health of males. Another study described that the blend prepared by *H. rosa sinensis* flower and *Maranta arundinacea L*. was used as a rice substitute owing to its low bulk density (0.83 g/mL) and starch digestibility (0.62 g/mL) as compared to rice (Antari et al. 2024). Diseases caused by oxidative stress are increasing globally, which can be managed by consuming antioxidant‐enriched products. Tea consumption is prevailing worldwide due to its soothing and relaxing properties. Herbal tea is formulated by combining green tea with *Ocimum gratissimum* , *Cymbopogon citratus* , *Cymbopogon flexuosus* , and *H. rosa sinensis*, and its antioxidant potential was evaluated by conducting DPPH and ABTS. The results depicted strong DPPH and ABTS scavenging activity by showing the respective EC50 values of 38.8 and 5.43 μg/mL, 53.6 and 11.6 μg/mL, 155.4 and 57.5 μg/mL, 295.3 and 49.1 μg/mL by *H. rosa sinensis*, *O. gratissimum* , *C. flexuosus* , and *C. citratus* , thereby could be used as a chemotherapeutic agent (Farooq and Sehgal 2019). Table 4 highlights the synergic role of *H. sinensis* with other medicinal plants.

**TABLE 4:** Synergistic studies of *Hibiscus rosa Sinensis* with other plants.

Disease	Plants	Supplementation and specimen	Outcomes	References
Diabetes mellitus	*Matricaria Chamomilla*	Peach drinks enriched with *H. rosa sinensis* and *Matricaria Chamomilla* , and rats	↑serum insulin, ↑HDL levels, ↓fasting blood glucose, ↓random blood glucose, ↓LDL, ↓TC, ↓TG	Yasin et al. (2025)
Antibacterial activity	*Chrysanthemum indicum* , and *Calendula officinalis* flower	Gram‐positive ( *Bacillus cereus* MTCC 430, *Staphylococcus aureus* MTCC 87, *Listeria monocytogenes* MTCC 657, *Clostridium perfringens* MTCC 450) and gram‐negative ( *Escherichia coli* MTCC 43, *Salmonella typhi* MTCC 1264, and *Pseudomonas aeruginosa* MTCC424) strains	MIC = 3.75%–7.5%, and MBC (minimum bactericidal concentration) = 1.9%–3.8%	Karnwal (2022)
Hepatic CYP3A4 activity	*Brassica oleracea* and *Tradescantia zebrina*	Rat liver microsomes	↑CYP3A4 activity, interact with aminopyrine metabolism	Han et al. (2020)
Anti‐inflammatory potential	*Hibiscus sabdariffa*	Endothelial cells (HUVECs) as a model for RAGE‐mediated inflammation screening	↓inflammation, ↓TNF‐α, ↓IL‐6, and ↓VCAM‐1	Lima (2022)
Wound healing activity	*Curcuma longa* rhizomes	Ointment prepared from *H. rosa sinensis* and *Curcuma longa* rhizome extracts, applied daily on Sprague Dawley rats for 20 days	↑wound contraction (93%)	Mustaffa et al. (2020)
	*Centella asiatica*	Combination application of *Centella asiatica* and *H. rosa sinensis* extract on 24 rats	↑wound healing, ↑COL53A, ↑CXCL11, ↑CSF2, ↑IL6ST, ↑CXCL5, ↑ITGA5, ↑PLAT, and ↑WISP1	Zulkurnain et al. (2025)
Body scrubber	*Citrofortunella macrocarpa* and *Psidium guajava*	*Citrofortunella macrocarpa*, *Psidium guajava* , and *H. rosa sinensis* extract prepared with sea salt, coconut oil, honey, and powdered milk	↓dry skin, ↓dead skin cells	Alfante et al. (2019)
Stress‐induced alopecia	*Baccaurea Racemosa*	*H. rosa‐sinensis L*. and *B. racemosa* extracts in male Wistar albino rats	↑hair length, ↑hair density, and ↑hair follicles	Indrayoni and Padmiswari (2022)

## 12 Molecular Docking

Molecular docking predicts how two molecules, such as a ligand and a protein, bind together by exploring conformations. It uses computational algorithms to estimate binding affinities, guiding drug design and virtual screening. Docking accelerates the identification of potential therapeutics by modeling intermolecular interactions and optimizing complementarity and structural precision. The current section contains molecular docking of various bioactive compounds of *H. sinensis* , like rutin, quercetin, and myricetin, against different proteins such as α‐glucosidase and superoxide dismutase (SOD) to evaluate the antidiabetic and hepatoprotective role of *H. sinensis* . The ligands, i.e., rutin (CID 5280805), quercetin (CID: 5280343), and myricetin (CID: 5281672), were accessed in 3D SDF format through the PubChem database. While, proteins, i.e., α‐glucosidase and SOD, were retrieved from Protein Data Bank (PDB) with the following PDB IDs 5DKZ (2.40 Å) and 1P7G (1.80 Å), in PDB format, respectively. Moreover, PyRx (AutoDock Vina) and Discovery Studio software were used to analyze binding affinity and visualize structures. The following grid box dimensions (X: 1.6825, Y: 129.1842, Z: 139,9902) for rutin (X: 0.0352, Y: 132.1277, Z: 159.8043) for quercetin, and (X: 122.2965, Y: 102.3505, Z: 25.0000) for myricetin and exhaustiveness (Akhtar et al. 2022) were observed during the docking of 5DKZ. While the 1P7G protein exhibited X: 190.4571, Y: 328.5863, Z: 19.0058 for rutin, X: 162.9047, Y: 329.2597, Z: 25.0000 for quercetin, and X: 141.6282, Y: 337.9629, Z: 25.0000 for myricetin, with exhaustiveness: 8.

**FIGURE 5:**
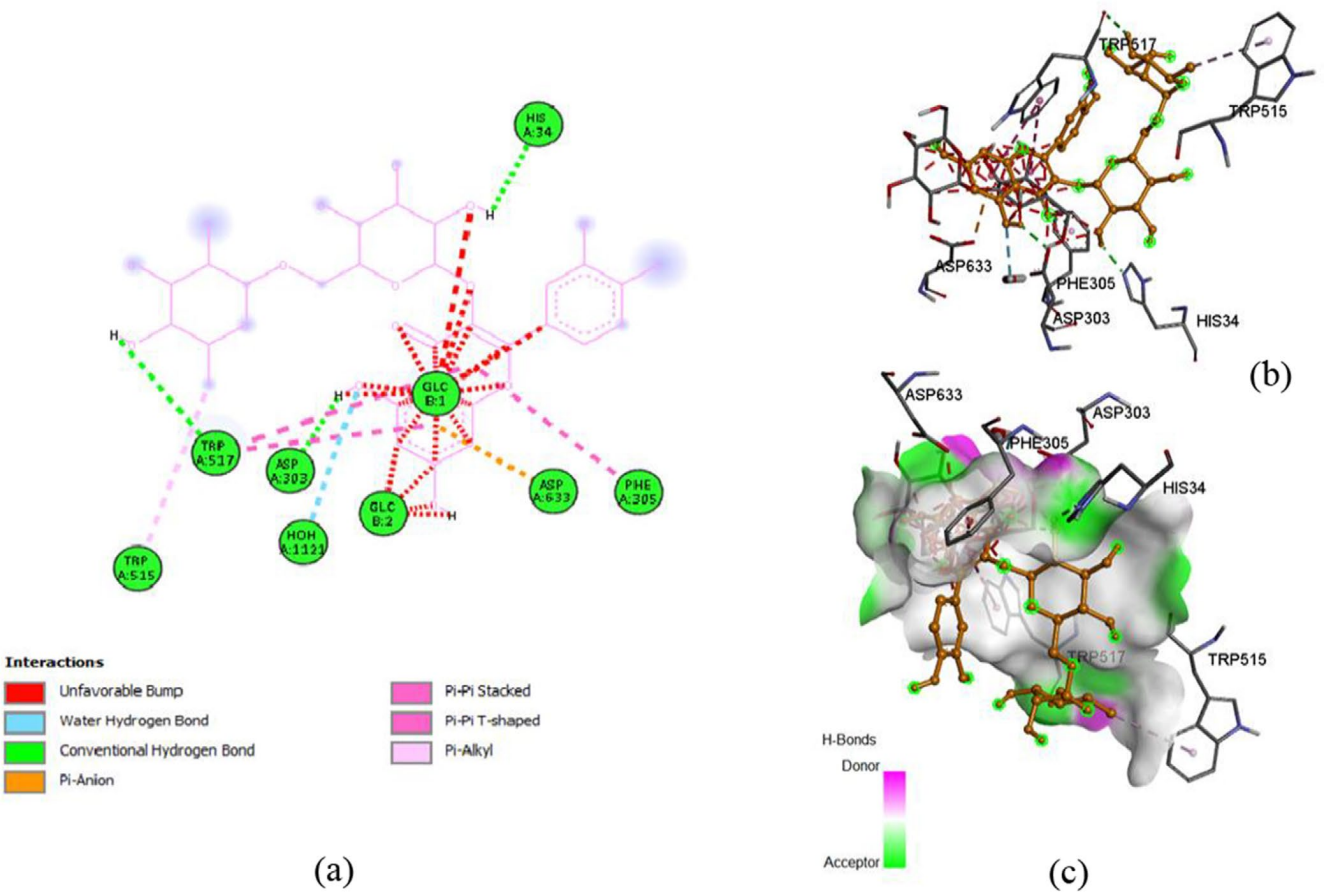
Rutin and α‐glucosidase interaction, (a) 2‐D structure, (b) 3‐D interaction, and (c) hydrogen bond surface.

**FIGURE 6:**
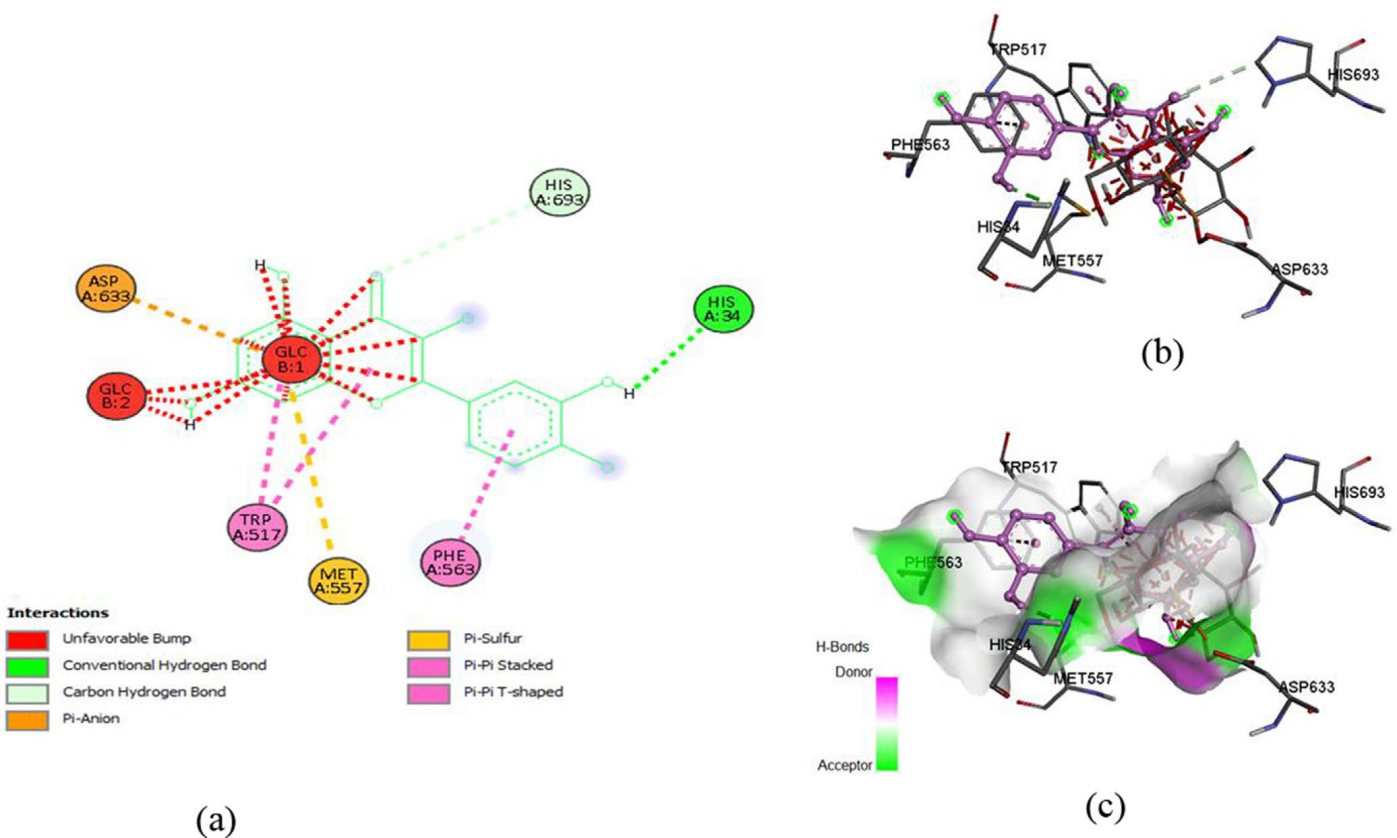
Quercetin and α‐glucosidase interaction, (a) 2‐D structure, (b) 3‐D interaction, and (c) hydrogen bond surface.

**FIGURE 7:**
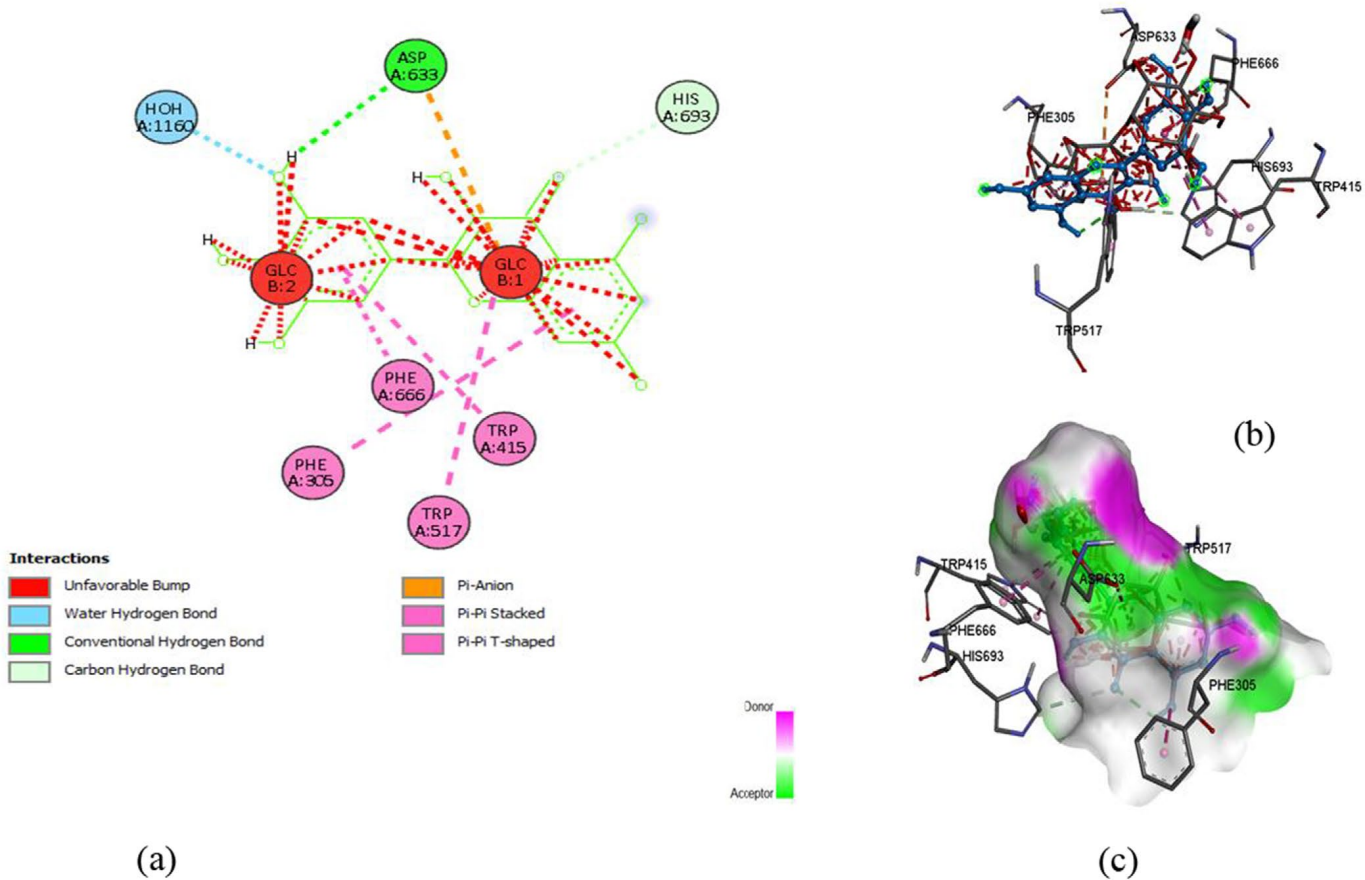
Myricetin and α‐glucosidase interaction, (a) 2‐D structure, (b) 3‐D interaction, and (c) hydrogen bond surface.

**FIGURE 8:**
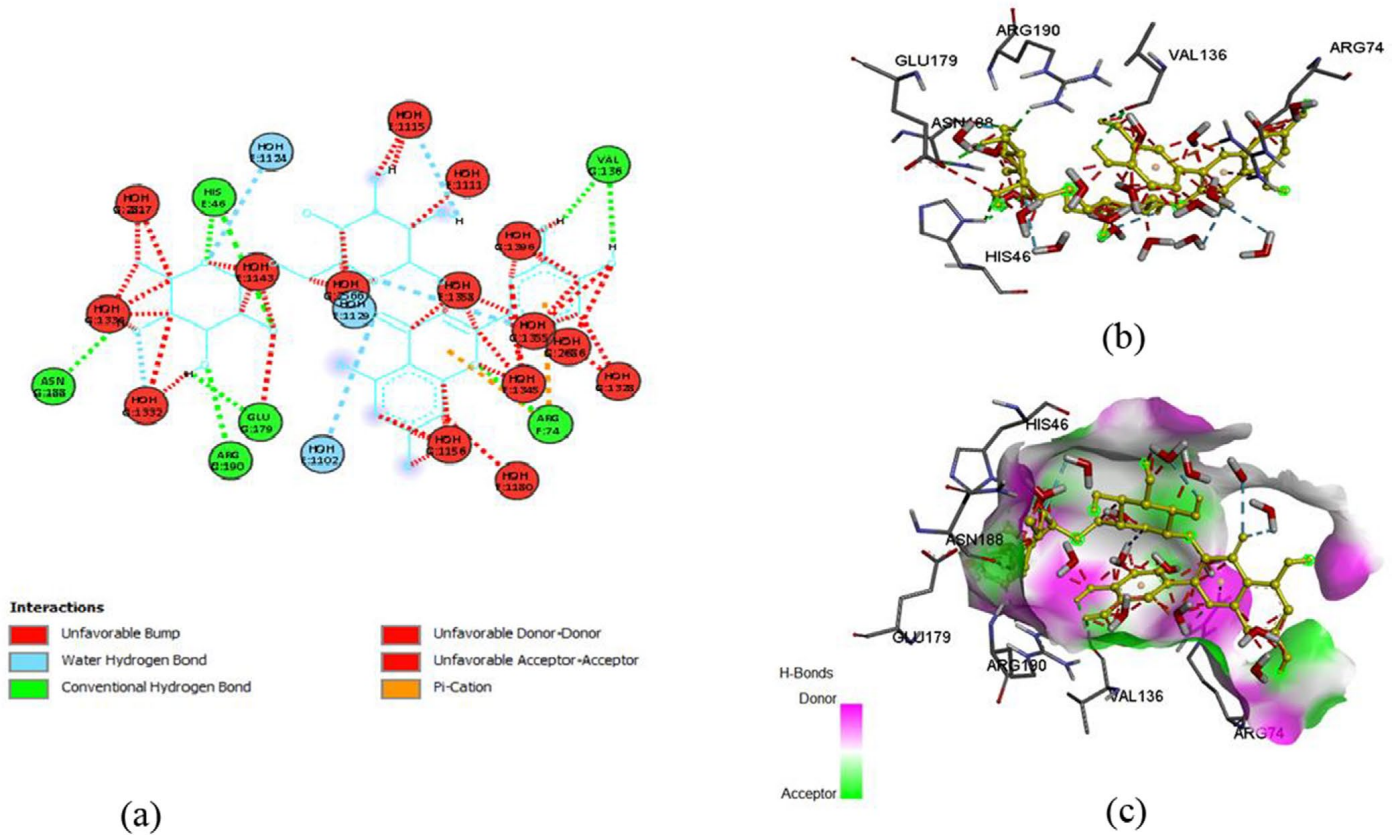
Rutin and SOD interaction, (a) 2‐D structure, (b) 3‐D interaction, and (c) hydrogen bond surface.

**FIGURE 9:**
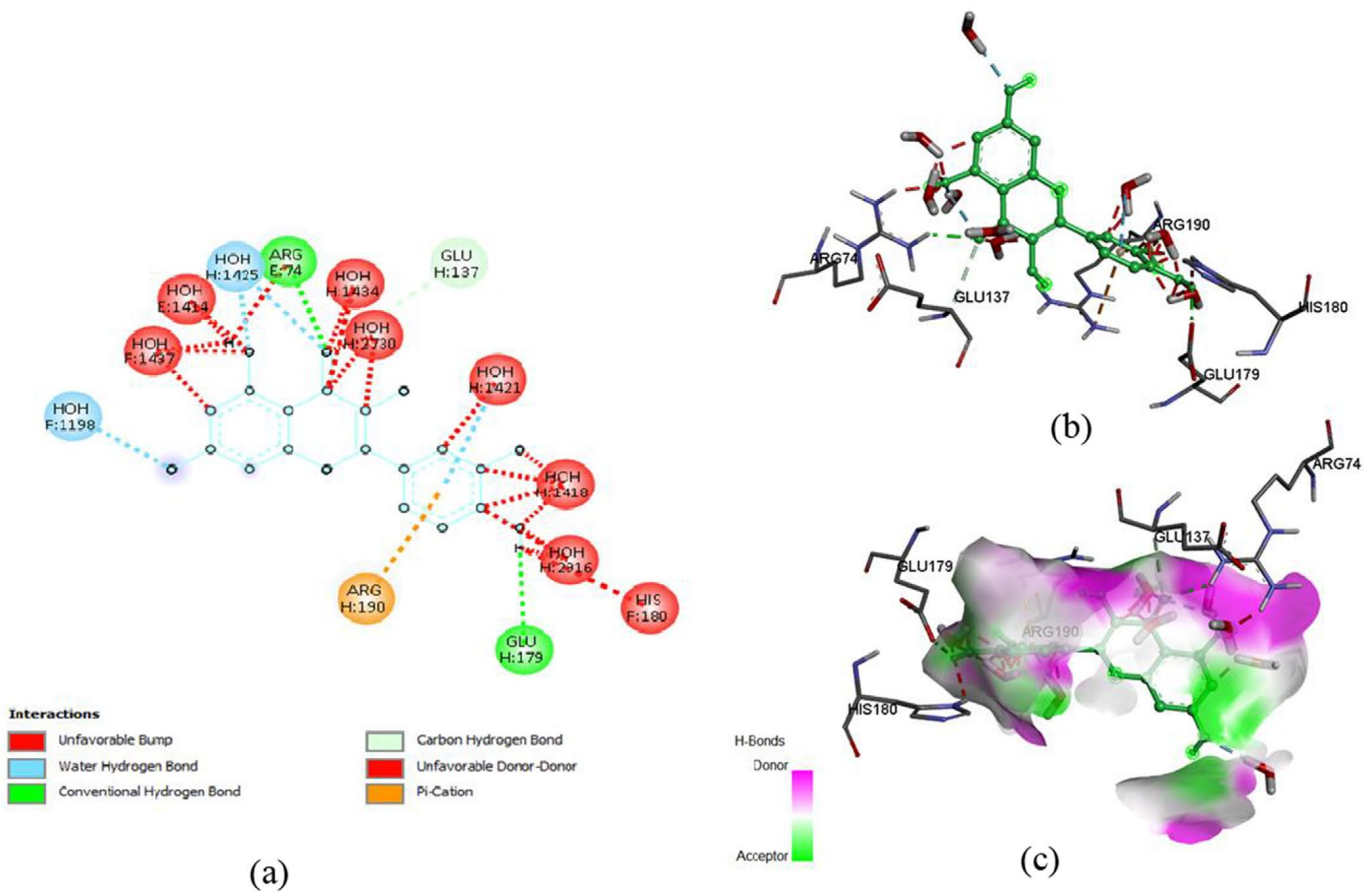
Quercetin and SOD interaction, (a) 2‐D structure, (b) 3‐D interaction, and (c) hydrogen bond surface.

**FIGURE 10:**
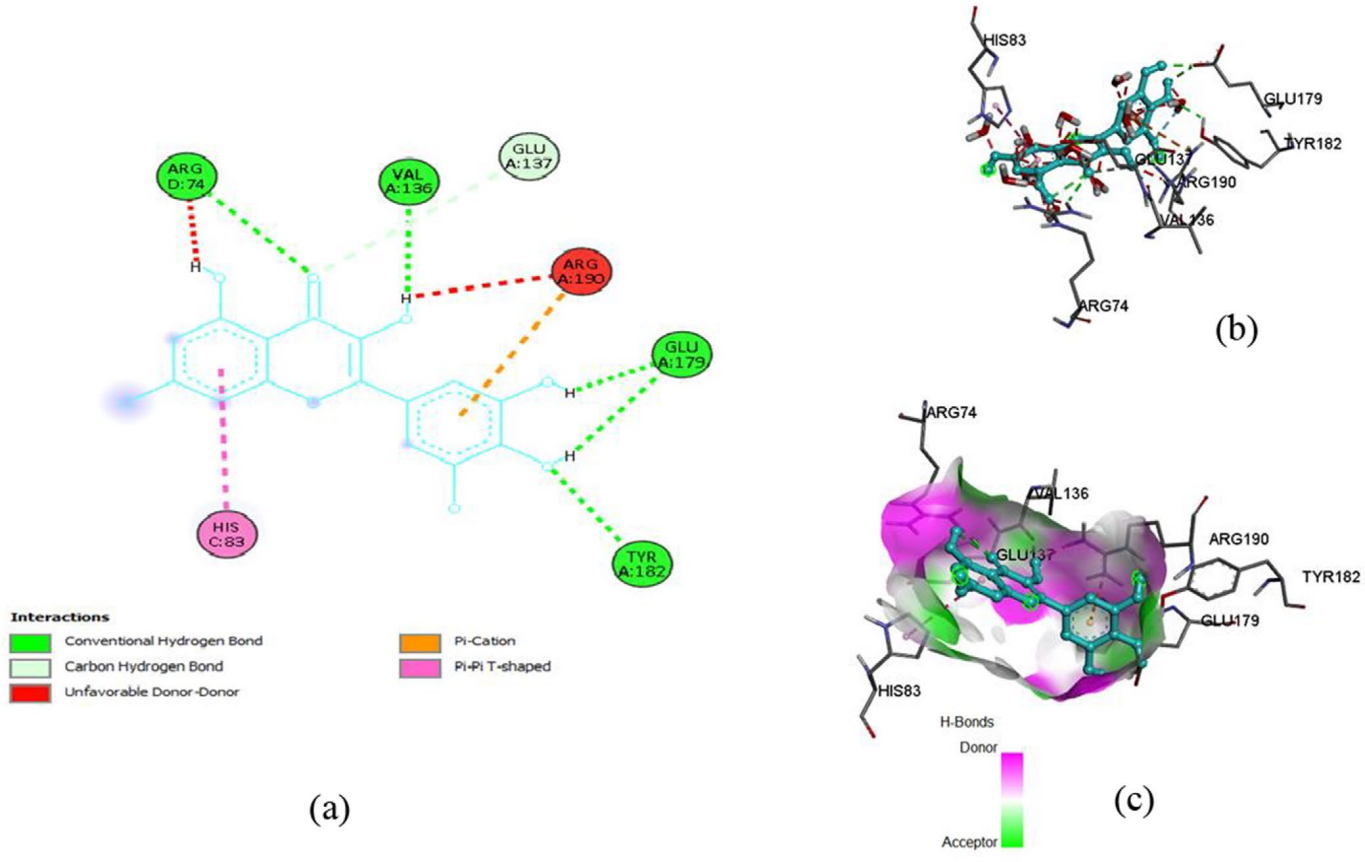
Myricetin and SOD interaction, (a) 2‐D structure, (b) 3‐D interaction, and (c) hydrogen bond surface.

The docking of ligands with proteins and their binding affinity is displayed in Figures 5, 6, 7, 8, 9, 10, as well as Tables 5 and 6.

**TABLE 5:** Residues with types of interaction and distance along with binding affinity of investigated molecules.

Protein	Compound	Amino acids	Distance (Å)	Category	Types	Energy (Kcal/mol)
α‐glucosidase	Rutin	ASP303	2.19926	Hydrogen Bond	Conventional Hydrogen Bond	−8.2
		HIS34	2.10947	Hydrogen Bond	Conventional Hydrogen Bond	
		TRP517	2.53231	Hydrogen Bond	Conventional Hydrogen Bond	
		ASP633	3.76315	Electrostatic	Pi‐Anion	
		PHE305	5.74975	Hydrophobic	Pi‐Pi Stacked	
		TRP515	4.96748	Hydrophobic	Pi‐Alkyl	
	Quercetin	HIS34	2.1427	Hydrogen Bond	Conventional Hydrogen Bond	
		HIS693	3.68684	Hydrogen Bond	Carbon Hydrogen Bond	−8
		ASP633	3.86745	Electrostatic	Pi‐Anion	
		MET557	5.33197	Other	Pi‐Sulfur	
		PHE563	4.02503	Hydrophobic	Pi‐Pi Stacked	
		TRP517	5.27173	Hydrophobic	Pi‐Pi T‐shaped	
	Myricetin	HIS693	3.65464	Hydrogen Bond	Carbon Hydrogen Bond	−8.6
		ASP633	4.39916	Electrostatic	Pi‐Anion	
		PHE305	5.43286	Hydrophobic	Pi‐Pi Stacked	
		TRP415	5.67302	Hydrophobic	Pi‐Pi Stacked	
		PHE666	4.8258	Hydrophobic	Pi‐Pi Stacked	

**TABLE 6:** Residues with types of interaction and distance along with binding affinity of investigated molecules.

Protein	Compound	Amino acids	Distance (Å)	Category	Types	Energy (Kcal/mol)
SOD	Rutin	HIS46	2.29024	Hydrogen Bond	Conventional Hydrogen Bond	−10.5
		ARG74	1.92328	Hydrogen Bond	Conventional Hydrogen Bond	
		ARG190	2.29096	Hydrogen Bond	Conventional Hydrogen Bond	
		GLU179	2.25398	Hydrogen Bond	Conventional Hydrogen Bond	
	Quercetin	ARG74	2.20032	Hydrogen Bond	Conventional Hydrogen Bond	−8.4
		ARG190	4.05366	Electrostatic	Pi‐Cation	
		GLU137	3.29044	Hydrogen Bond	Carbon Hydrogen Bond	
		GLU179	2.19554	Hydrogen Bond	Conventional Hydrogen Bond	
	Myricetin	ARG190	4.25339	Electrostatic	Pi‐Cation	−8.3
		HIS83	4.81584	Hydrophobic	Pi‐Pi T‐shaped	
		GLU137	3.66604	Hydrogen Bond	Carbon Hydrogen Bond	
		ARG74	2.40734	Hydrogen Bond	Conventional Hydrogen Bond	
		TRY182	2.44405	Hydrogen Bond	Conventional Hydrogen Bond	

**FIGURE 11:**
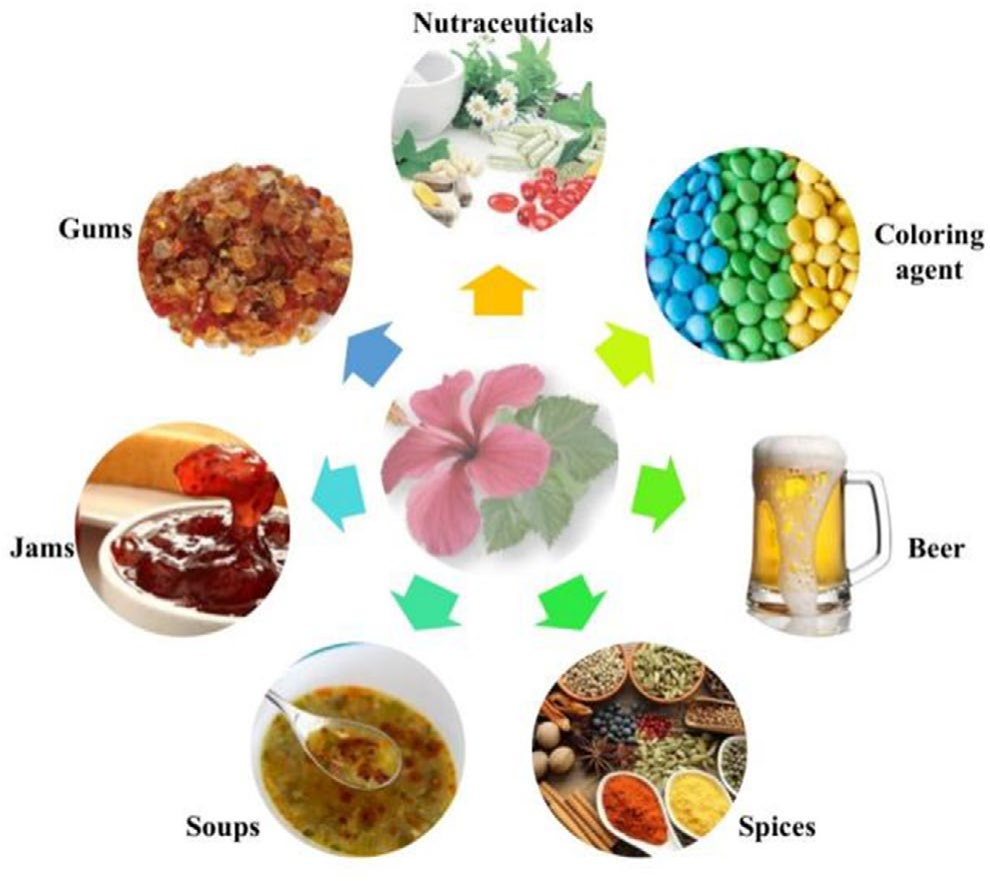
Industrial applications of *H. rosa‐sinensis* .

## 13 Safety and Toxicity

The safety of plants and compounds is a fundamental consideration in the healthcare and food industries before their implementation in markets. Excessive and long‐term consumption of compounds poses several adverse health consequences in the form of carcinogens, cognitive impairment, hepatotoxicity, renal failure, and dermatological and gastrointestinal disorders. Limited investigations regarding the safety of plants and their derived compounds are conducted, which provide limitations in their implementations. Regarding the safety of *H. rosa‐sinensis* , it is safe to use and even effective in curing various prevailing health disorders among individuals. Meena et al. (2014) investigated the acute toxicity of the methanolic flower extract of *H. rosa‐sinensis* in Balb/c mice by providing different doses, i.e., 100, 200, 400, 800, and 1600 mg/kg body weight of extract through oral gavage. It has been observed that the methanolic flower extract did not produce any adverse effects in the form of behavior abnormality, respiratory symptoms, reduced food and water consumption, and hair loss up to 800 mg/kg body weight. However, ~20% mortality cases were observed in Balb/c mice consuming 1600 mg/kg *H. rosa‐sinensis* extract.

Furthermore, Valdivié and Martínez (2022) and Nath and Yadav (2014) revealed the toxicity of the methanolic leaves extract of *H. rosa‐sinensis* in mice, which were supplemented with an 800 mg/kg body weight dose for 2 weeks. The elevated ALT, ALP, AST, urea, creatinine, and bilirubin levels were observed at the 800 mg/kg body weight dose. However, the extract's safe and lethal dose (LD50) was recorded up to the doses of 400 and 2000 mg/kg body weight, respectively. Recently, a study on albino rats revealed that the LD50 for the methanolic extract of the stem bark of *H. sinensis* was 5000 mg/kg, showing its acceptance and tolerability at such a high dose (Umar et al. 2024). Considering all this, it has been verified that *H. sinensis* could be safe up to 400 mg/kg body weight for 14 days; however, 800 mg/kg for 14 days might cause hepato‐renal adverse effects. Moreover, the LD50 for the leaves extract is 2000 mg/kg, while for the stem extract it is 5000 mg/kg.

## 14 Industrial Applications

Industries have been utilizing natural resources for the last few years to develop sustainable and eco‐friendly environmental products that are cost‐effective and healthy for consumers. Medicinal plants have gained importance in various pharmaceutical, nutraceutical, cosmetic, food and beverage industries. *H. rosa‐sinensis* has found its applications in various sectors due to the presence of unique bioactive compounds. A study by Khan et al. (2017) and Baranova et al. (2012) reported that *H. rosa‐sinensis* has been employed as a flavoring agent in preparing jams, soups, and spices. Another study by Pieracci et al. (2021) employed *H. rosa‐sinensis* flowers in the beer industry. They evaluated its aroma and sensory properties, which revealed the floral and fruity notes enriched with esters and alcohol in Hibiscus beer. At the same time, the control beer had malty and hoppy notes. Additionally, the red color of *H. rosa‐sinensis* is fundamental owing to the presence of anthocyanins, which are used as coloring agents in food industries along with cyanidin‐3‐*O*‐glucoside and delphinidin (Mejía et al. 2023; Sinha and Asimi 2007).

The antioxidant, anticancer, hypoglycemic, hepatoprotective, hypotensive, and anti‐depressive attributes of *H. rosa‐sinensis* enable it to synthesize drugs and supplements in the nutraceutical and pharmaceutical industries. The polymers of gums and mucilage are prepared for *H. rosa‐sinensis* , which are employed in drug preparation and enhance their bioavailability (Yahaya et al. 2023; Weerasingha et al. 2021). Anthocyanins are extracted in abundance by preparing a methanolic solution of 4% citric acid, which is then used as a natural dye by maintaining a standard pH of 4. However, cotton and silk dyes' rapid properties were achieved with stannous mordanted fabrics (Vankar and Shukla 2011; Vankar and Srivastava 2008). The industrial applications of *H. rosa‐sinensis* are displayed in Figure 11.

## 15 Conclusion and Future Perspectives

Due to versatile biomedical applications contributing significantly to health maintenance, there has been an increased demand for improving the endurance and subsistence of individuals through medicinal plants, as well as *H. rosa‐sinensis* . The bioactive compounds, such as quercetin, kaempferol, anthocyanins, saponins, alkaloids, and essential oils, enhance the therapeutic potential of *H. rosa‐sinensis* . These compounds reduce oxidative and oxidative stress‐induced disorders by attenuating free radicals and inflammatory markers. Various in vitro and in vivo studies have confirmed the antidiabetic, hypolipidemic, hypotensive, neuroprotective, and cardioprotective properties. Moreover, the anticancer activity of *H. rosa‐sinensis* via modulation of inflammatory markers (IL‐6, IL‐1β, TNF‐α) and genes like ESR1 and HER2 has also been reported. Studies have also reported the safety of *H. rosa‐sinensis* in nutraceuticals, supplements, and functional foods, with few adverse impacts. Its adaptogenic ability to diverse environments makes it an excellent choice for industrial applications. Its flowers and plant extracts are used as a coloring and flavoring agent in food industries and as a tonic to slow aging and promote hair growth. Despite its substantial pharmaceutical and nutraceutical applications there is a lack of clinical trials on safety and therapeutic potential, thus hindering its significance and future implications. Moreover, *H. rosa‐sinensis* contains diverse bioactive compounds like anthocyanins, which are sensitive and unstable towards light, temperature, pH, and even oxygen. Therefore, further clinical investigations are required to validate its efficacy, extraction methods, and industrial applications. In addition, the handling of this plant needs special attention and effective techniques to obtain its maximum bioactives yield. In the last, the synergistic interaction with other plants, compounds, standard drugs, and therapeutic approaches can enhance *H. rosa‐sinensis* pharmacological potential.

## Author Contributions

**Hassan Raza:** writing – original draft. **Muhammad Tauseef Sultan:** supervision. **Khalil Ahmad:** supervision. **Muhammad Maaz:** writing – original draft. **Shehnshah Zafar:** writing – reviewing. **Ahmad Mujtaba Noman:** writing – review and editing. **Entessar Mohammad Al Jbawi:** writing – review and editing.

## Funding

The authors have nothing to report.

## Ethics Statement

The authors have nothing to report.

## Consent

All authors are agreed to publish this work.

## Conflicts of Interest

The authors declare no conflicts of interest.

## Data Availability

The data that support the findings of this study are available from the corresponding author upon reasonable request.

## References

[fsn371254-bib-0001] Abate, T. A. , and A. N. Belay . 2022. “Assessment of Antibacterial and Antioxidant Activity of Aqueous Crude Flower, Leaf, and Bark Extracts of Ethiopian *Hibiscus rosa‐sinensis* Linn: Geographical Effects and Co2Res2/Glassy Carbon Electrode.” International Journal of Food Properties 25, no. 1: 1875–1889.

[fsn371254-bib-0002] Abd El‐Kader, M. F. H. , M. T. Elabbasy , A. T. Adeboye , M. G. Zeariya , and A. A. Menazea . 2021. “Morphological, Structural and Antibacterial Behavior of Eco‐Friendly of ZnO/TiO2 Nanocomposite Synthesized via *Hibiscus rosa‐sinensis* Extract.” Journal of Materials Research and Technology 15: 2213–2220.

[fsn371254-bib-0003] Adil, E. H. , and N. Manampiring . 2020. “Hepatoprotective Activities of Polar and Non‐ Polar Extract Kembang Sepatu Flower (*Hibiscus Rosasinensis* L.).” Indonesian Biodiversity 1: 50–59.

[fsn371254-bib-0004] Afiune, L. A. F. , T. Leal‐Silva , Y. K. Sinzato , et al. 2017. “Beneficial Effects of *Hibiscus rosa‐sinensis* L. Flower Aqueous Extract in Pregnant Rats With Diabetes.” PLoS One 12: e0179785. 28644857 10.1371/journal.pone.0179785 PMC5482446

[fsn371254-bib-0005] Agarwal, S. , and R. Prakash . 2013. “Essential Oil Composition of Solvent Extract of Hibiscus Rosasinensis Flower.” Oriental Journal of Chemistry 29, no. 2: 813–814.

[fsn371254-bib-0006] Agrawal, K. K. , Y. Murti , N. Agrawal , and T. Gupta . 2021. “In Silico Studies of Bioactive Compounds From *Hibiscus rosa‐sinensis* Against HER2 and ESR1 for Breast Cancer Treatment.” International Journal of Pharmaceutical Sciences and Nanotechnology (IJPSN) 14, no. 6: 5665–5671.

[fsn371254-bib-0007] Ahmed, N. , M. A. Sheikh , M. Ubaid , P. Chauhan , K. Kumar , and S. Choudhary . 2024. “Comprehensive Exploration of Marine Algae Diversity, Bioactive Compounds, Health Benefits, Regulatory Issues, and Food and Drug Applications.” Measurement: Food 14: 100163.

[fsn371254-bib-0008] Akhtar, S. , S. M. Asiri , F. A. Khan , et al. 2022. “Formulation of Gold Nanoparticles With Hibiscus and Curcumin Extracts Induced Anti‐cancer Activity.” Arabian Journal of Chemistry 15, no. 2: 103594.

[fsn371254-bib-0009] Al‐Alak, S. K. , R. M. S. AL‐Oqaili , B. B. Mohammed , and N. Abd‐Alkhalik . 2015. “Antibacterial Activity of *Hibiscus rosa‐sinensis* Extract and Synergistic Effect With Amoxicillin Against Some Human Pathogens.” American Journal of Phytomedicine and Clinical Therapeutics 3: 20–27.

[fsn371254-bib-0010] AL‐Azawi, R. S. , and F. N. Al‐hady . 2020. “Testosterone and Progesterone Levels, Gene Expression of Androgen and Progesterone Receptors in Albino Male Rats Treated With Phenolic Flower Extract of *Hibiscus Rosa‐sinensis* L.” Medico‐Legal Update 20: 1.

[fsn371254-bib-0011] Alfante, K. C. A. , G. F. Suratos , and K. L. Dayrit . 2019. “Synergistic Activity of Calamansi (Citrofortunella Microcarpa), Riped Guava ( *Psidium guajava* ) and Gumamela ( *Hibiscus rosa‐sinensis* ) Flower Extracts as Body Scrub.” Philippine Journal of Natural and Social Sciences 1: 1.

[fsn371254-bib-0012] Al‐Snafi, A. E. 2018. “Chemical Constituents, Pharmacological Effects and Therapeutic Importance of *Hibiscus rosa‐sinensis*‐A Review.” IOSR Journal of Pharmacy 8, no. 7: 101–119.

[fsn371254-bib-0013] Amtaghri, S. , A. Amssayef , M. Slaoui , and M. Eddouks . 2022. “Antihypertensive and Vasorelaxant Effects of *Hibiscus rosa‐sinensis* Through Angiotensin‐Converting Enzyme‐2 (ACE‐2), and Ca2+ Channels Pathways.” Cardiovascular & Haematological Disorders‐Drug Targets (Formerly Current Drug Targets‐Cardiovascular & Hematological Disorders) 22, no. 1: 27–37. 10.2174/1871529X22666220329190331 35352670

[fsn371254-bib-0014] Amtaghri, S. , A. Qabouche , M. Slaoui , and M. Eddouks . 2024. “A Comprehensive Overview of *Hibiscus rosa‐sinensis* L.: Its Ethnobotanical Uses, Phytochemistry, Therapeutic Uses, Pharmacological Activities, and Toxicology.” Endocrine, Metabolic & Immune Disorders 24, no. 1: 86–115. 10.2174/1871530323666230522113405 37218183

[fsn371254-bib-0015] Angelopoulou, E. , Y. N. Paudel , S. G. Papageorgiou , and C. Piperi . 2022. “Environmental Impact on the Epigenetic Mechanisms Underlying Parkinson's Disease Pathogenesis: A Narrative Review.” Brain Sciences 12, no. 2: 175. 35203939 10.3390/brainsci12020175 PMC8870303

[fsn371254-bib-0016] Ansari, P. , S. Azam , J. M. A. Hannan , P. R. Flatt , and Y. H. A. Wahab . 2020. “Anti‐Hyperglycaemic Activity of *H. rosa‐sinensis* Leaves Is Partly Mediated by Inhibition of Carbohydrate Digestion and Absorption, and Enhancement of Insulin Secretion.” Journal of Ethnopharmacology 253: 112647. 32035878 10.1016/j.jep.2020.112647

[fsn371254-bib-0017] Antari, A. L. , I. Saraswati , E. Annisa , and A. A. Fatma . 2024. “Formulation and Characterization of Analog Rice Using Arrowroot (*Maranta arundinacea* L.) and Hibiscus Flower (*Hibiscus rosa Sinensis* L.).” Pharmacy & Pharmaceutical Sciences Journal/Jurnal Farmasi Dan Ilmu Kefarmasian Indonesia 11, no. 3: 386–394.

[fsn371254-bib-0018] Anuradha, C. T. , and P. Raji . 2019. “Effect of Annealing Temperature on Antibacterial, Antifungal and Structural Properties of Bio‐Synthesized Co3O4 Nanoparticles Using *Hibiscus Rosa‐sinensis* .” Materials Research Express 6, no. 9: 095063.

[fsn371254-bib-0019] Arullappan, S. , Z. Zakaria , and D. F. Basri . 2009. “Preliminary Screening of Antibacterial Activity Using Crude Extracts of Hibiscus rosa Sinensis.” Tropical Life Sciences Research 20, no. 2: 109–118. 24575183 PMC3819052

[fsn371254-bib-0020] Aziz, M. A. , S. Z. Raduan , A. H. Roslida , Z. A. Zakaria , A. Zuraini , and M. N. Hakim . 2021. “Anti‐Pyretic Activity of Two Varieties of *Hibiscus rosa Sinensis* L.” Biomedical and Pharmacology Journal 14, no. 1: 61–74.

[fsn371254-bib-0021] Bala, R. , R. Kaur , B. Kaur , and P. Kaur . 2022. “ *Hibiscus rosa Sinensis* Linn.: A Phytochemical and Pharmacological Review.” International Journal of Health Sciences 6: 5165–5193.

[fsn371254-bib-0022] Baranova, V. S. , I. F. Rusina , D. A. Guseva , N. N. Prozorovskaya , O. M. Ipatova , and O. T. Kasaikina . 2012. “The Antiradical Activity of Plant Extracts and Healthful Preventive Combinations of These Exrtacts With the Phospholipid Complex.” Biomeditsinskaya Khimiya 58, no. 6: 712–726. 10.18097/pbmc20125806712 23350203

[fsn371254-bib-0023] Begum, Z. , and I. Younus . 2018. “Hibiscus rosa Sinensis Mediate Anxiolytic Effect via Modulation of Ionotropic GABA‐A Receptors: Possible Mechanism of Action.” Metabolic Brain Disease 33: 823–827. 29372452 10.1007/s11011-018-0188-4

[fsn371254-bib-0024] Begum, Z. , I. Younus , and H. Khan . 2018. “Analgesic and Anti‐Inflammatory Activities of the Ethanol Extract of *Hibiscus rosa Sinensis* Linn (Roots).” Pakistan Journal of Pharmaceutical Sciences 31, no. 5: p1927. 30150191

[fsn371254-bib-0025] Bhaskar, A. , and V. G. Vidhya . 2012. “Hypoglycemic and Hypolipidemic Activity of *Hibiscus rosa* Sinensis Linn on Streptozotocin–Induced Diabetic Rats.” International Journal of Diabetes in Developing Countries 32: 214–218.

[fsn371254-bib-0026] Biswas, A. , U. J. D'Souza , S. Bhat , and D. Damodar . 2014. “The Hepatoprotective Effect of *Hibiscus rosa Sinensis* Flower Extract on Diet‐Induced Hypercholesterolemia in Male Albino Wister Rats.” International Journal of Pharma Medicine and Biological Sciences 4, no. 6: 1–10.

[fsn371254-bib-0027] Buarki, F. , H. AbuHassan , F. Al Hannan , and F. Z. Henari . 2022. “Green Synthesis of iron Oxide Nanoparticles Using *Hibiscus rosa Sinensis* Flowers and Their Antibacterial Activity.” Journal of Nanotechnology 2022, no. 1: 5474645.

[fsn371254-bib-0028] Castillo‐Rangel, C. , G. Marin , K. A. Hernandez‐Contreras , and M. M. Vichi‐Ramirez . 2023. “Neuroinflammation in Parkinson's Disease: From Gene to Clinic: A Systematic Review.” International Journal of Molecular Sciences 24, no. 6: 5792. 36982866 10.3390/ijms24065792 PMC10051221

[fsn371254-bib-0029] Chaachouay, N. , and L. Zidane . 2024. “Plant‐Derived Natural Products: A Source for Drug Discovery and Development.” Drugs and Drug Candidates 3, no. 1: 184–207.

[fsn371254-bib-0030] Chai, Y. T. 2020. Antioxidant and Antibacterial Activities of Hybrid, White and Orange Hibiscus (Hibiscus Rosa‐Sinensis). Tunku Abdul Rahman University College.

[fsn371254-bib-0031] Chauhan, K. , and S. Rani . 2024a. “Evaluation of Antidiabetic Potential of Hibiscus rosa Sinensis on Streptozotocin‐Induced Diabetes on Wistar Albino Rats.” Journal of Applied and Natural Science 16, no. 1: 245–250.

[fsn371254-bib-0032] Chauhan, K. , and S. Rani . 2024b. “Synergistic Effect of Camel Milk and *Hibiscus rosa Sinensis* on the Reproduction of Type II Diabetic Albino Wistar Rats.” Journal of Applied and Natural Science 16, no. 4: 1582.

[fsn371254-bib-0033] Chen, Z. , W. Ding , X. Yang , T. Lu , and Y. Liu . 2024. “Isoliquiritigenin, a Potential Therapeutic Agent for Treatment of Inflammation‐Associated Diseases.” Journal of Ethnopharmacology 318: 117059. 37604329 10.1016/j.jep.2023.117059

[fsn371254-bib-0034] Daud, D. , N. F. M. Arsad , A. Ismail , and A. Tawang . 2016. “Anti‐Pyretic Action of *Caulerpa lentillifera*, Hibiscus rosa‐Sinensis and Piper Sarmentosum Aqueous Extract in Mice.” Asian Journal of Pharmaceutical and Clinical Research 9, no. 1: 9–11.

[fsn371254-bib-0035] Dayal, R. , B. Kumar , I. Melkani , et al. 2022. “Hepatoprotective Potential of Aqueous Extract of Hibiscus Rosasinensis and *Butea monosperma* Against Fe‐NTA Induced Hepatotoxicity in Rats.” Research Journal of Pharmacy and Technology 15, no. 7: 3213–3220.

[fsn371254-bib-0036] De Boer, H. J. , and C. Cotingting . 2014. “Medicinal Plants for Women's Healthcare in Southeast Asia: A Meta‐Analysis of Their Traditional Use, Chemical Constituents, and Pharmacology.” Journal of Ethnopharmacology 151, no. 2: 747–767. 24269772 10.1016/j.jep.2013.11.030

[fsn371254-bib-0037] Dolgacheva, L. P. , V. P. Zinchenko , and N. V. Goncharov . 2022. “Molecular and Cellular Interactions in Pathogenesis of Sporadic Parkinson Disease.” International Journal of Molecular Sciences 23, no. 21: 13043. 36361826 10.3390/ijms232113043 PMC9657547

[fsn371254-bib-0038] Dowara, M. , I. Gogoi , and S. Saikia . 2024. “Phytochemical Screening and In Vitro Antibacterial Activity of *Hibiscus rosa‐sinensis* L. Leaf Extracts.” Asian Journal of Biological and Life Sciences 13, no. 1: 35–39.

[fsn371254-bib-0039] Elemike, E. E. , D. C. Onwudiwe , and J. I. Mbonu . 2021. “Facile Synthesis of Cellulose–ZnO‐Hybrid Nanocomposite Using *Hibiscus rosa‐sinensis* Leaf Extract and Their Antibacterial Activities.” Applied Nanoscience 11, no. 4: 1349–1358.

[fsn371254-bib-0040] El‐Sayed, M. S. S. 2018. “Effect of Hepatoprotective Role Evaluation of *hibiscus rosa‐sinensis* Leaves and Pomegranate (*punica granatum*) Peels Aq Ueous Extracts on Male Albino Rats.” Zagazig Journal of Agricultural Research 45, no. 1: 349–362.

[fsn371254-bib-0041] Eze, I. , and D. Nwibo . 2017. “Effects of Processing on Proximate Composition of *Hibiscus rosa‐sinensis* Leaf.” European Journal of Medicinal Plants 18, no. 2: 1–13.

[fsn371254-bib-0042] Falade, O. S. , M. A. Aderogba , O. Kehinde , B. A. Akinpelu , B. O. Oyedapo , and S. R. Adewusi . 2009. “Studies on the Chemical Constituents, Antioxidants and Membrane Stability Activities of *Hibiscus rosa Sinensis* .” Nigerian Journal of Natural Products Medicine 13: 58–64.

[fsn371254-bib-0043] Farasayu, R. D. , M. W. Rachmawati , I. D. Ana , A. Syaify , and D. Listyarifah . 2021. “The Effect of Hibiscus Flower Extract ( *Hibiscus rosa‐sinensis* L.) on the Growth of *Streptococcus sanguinis* Bacteria.” In BIO Web of Conferences, vol. 41, p07006. EDP Sciences.

[fsn371254-bib-0044] Farooq, S. , and A. Sehgal . 2019. “Scrutinizing Antioxidant Interactions Between Green Tea and Some Medicinal Plants Commonly Used as Herbal Teas.” Journal of Food Biochemistry 43, no. 9: e12984. 31489661 10.1111/jfbc.12984

[fsn371254-bib-0045] Gauthaman, K. K. , M. T. Saleem , P. T. Thanislas , et al. 2006. “Cardioprotective Effect of the *Hibiscus rosa Sinensis* Flowers in an Oxidative Stress Model of Myocardial Ischemic Reperfusion Injury in Rat.” BMC Complementary and Alternative Medicine 6: 1–8. 16987414 10.1186/1472-6882-6-32 PMC1592511

[fsn371254-bib-0046] Geeganage, J. R. , and M. D. T. L. Gunathilaka . 2024. “Mechanistic Insight Into Anti‐Inflammatory Potential of *Hibiscus rosa‐sinensis* Flower Extract as a Herbal Remedy: A Systematic Review.” Journal of Herbal Medicine 45: 100884.

[fsn371254-bib-0047] Ghadigaonkar, S. 2023. “Evaluation of Antibacterial Activity of Ethanolic and Aqueous Leaf Extract of *Hibiscus rosa‐sinensis* in Mice.” Group 4: 6.

[fsn371254-bib-0048] Ghaffar, F. R. A. , and I. A. El‐Elaimy . 2012. “In Vitro, Antioxidant and Scavenging Activities of *Hibiscus rosa Sinensis* Crude Extract.” Journal of Applied Pharmaceutical Science 2: 151–158.

[fsn371254-bib-0049] Goldberg, K. H. , A. C. Yin , A. Mupparapu , E. P. Retzbach , G. S. Goldberg , and C. F. Yang . 2016. “Components in Aqueous *Hibiscus rosa‐sinensis* Flower Extract Inhibit In Vitro Melanoma Cell Growth.” Journal of Traditional and Complementary Medicine 7, no. 1: 45–49. 28053887 10.1016/j.jtcme.2016.01.005 PMC5198834

[fsn371254-bib-0050] Gomathi, N. , T. Malarvili , R. Mahesh , and V. H. Begum . 2008. “Lipids Lowering Effect of *Hibiscus rosa‐sinensis* Flower Petals on Monosodium Glutamate (MSG) Induced Obese Rats.” Pharmacology 1: 400–409.

[fsn371254-bib-0051] Goyal, S. , D. Thirumal , S. Singh , et al. 2025. “Basics of Antioxidants and Their Importance.” In Antioxidants: Nature's Defense Against Disease, 1–20. Purdue University.

[fsn371254-bib-0052] Guddeti, V. , D. M. Babu , B. Nagamani , M. R. Teja , M. S. Sravani , and C. H. Spandana . 2015. “Evaluation of Anti Inflammatory Activity of hibiscus rosa Sinensis Linn. Flower Extract in Rats.” International Journal of Pharmaceutical, Chemical & Biological Sciences 5: p532.

[fsn371254-bib-0053] Gulati, K. 2022. “Evaluation of Cellular and Molecular Mechanisms of Therapeutic Effects of Hibiscus Rosa‐Sinensis and *Piper nigrum* in Experimental Model of Bronchial Asthma.” Medicon Pharmaceutical Sciences 2: 2–13.

[fsn371254-bib-0054] Gupta, A. , G. K. Jana , M. S. Jangdey , and D. Soni . 2014. “Exploration of Anti‐Inflammatory Activity of Aquaeous Extract of Hibiscus rosa Sinesis (Malvacae).” Research Journal of Pharmacognosy and Phytochemistry 6, no. 3: 109–111.

[fsn371254-bib-0056] Han, C. J. , G. A. Akowuah , M. S. A. Shukkoor , and A. Biswas . 2020. “Modulation of Rat Hepatic CYP3A4 Activity by *Brassica oleracea*, *Hibiscus rosa Sinensis*, and *Tradescantia zebrina* .” Biointerface Research in Applied Chemistry 11, no. 1: 7453–7459.

[fsn371254-bib-0057] Harini, V. S. 2024. “Evaluation of the Anticancer, Antidiabetic, and In Vitro Wound Healing Properties of the Aqueous and Ethanolic Extract of *Hibiscus rosa‐sinensis* L.” Journal of Pharmacy & Bioallied Sciences 16, no. 2: S1217–S1222. 38882727 10.4103/jpbs.jpbs_545_23 PMC11174197

[fsn371254-bib-0058] Hussain, N. , M. N. Chaudhary , A. A. Anjum , et al. 2017. “Amelioration of Hepato‐Renal Toxicity by a Flavonoid‐Rich Fraction of *Hibiscus Rosa‐Sinenses* (Leaves and Flowers) in Male Rabbits Intoxicated by Cadmium.” Polish Journal of Environmental Studies 26, no. 4: 1551–1563.

[fsn371254-bib-0059] Indrayoni, P. , and A. A. I. M. Padmiswari . 2022. “Potential of *Hibiscus Rosa‐sinensis* L. and *Baccaurea Racemosa* Extract as a Hair Growth With Tail Suspension Test Stress‐Induced Alopecia.” Ad‐Dawaa'journal of Pharmaceutical Sciences 5, no. 1: 28–33.

[fsn371254-bib-0060] Iqbal, A. , and M. Z. U. Rehman . 2023. “Determining Genetic Variability and Taxonomy of *Hibiscus rosa‐sinensis* Through rbcL Molecular Marker: Genetic Variability and Taxonomy of *Hibiscus rosa‐sinensis* .” Pakistan BioMedical Journal 6, no. 6: 29–36.

[fsn371254-bib-0061] Jana, T. K. , S. Das , A. Ray , D. Mandal , S. Giri Jana , and J. Bhattacharya . 2013. “Study of the Effects of Hibiscusrosa‐Sinensis Flower Extract on the Spermatogenesis of Male Albino Rats.” Journal of Physiology and Pharmacology Advances 3, no. 6: 167–171.

[fsn371254-bib-0062] Jeevan Kumar, G. J. , and S. Rajeshkumar . 2022. “Rose Hibiscus Black Currant Tea Extract Mediated Silver Nanoparticles and Its Anti‐Microbial, Anti‐Oxidant, Anti‐Inflammatory and Cytotoxic Effect.”

[fsn371254-bib-0063] Johariya, V. , A. Joshi , N. Malviya , and S. Malviya . 2024. “Introduction to Cancer.” In Medicinal Plants and Cancer Chemoprevention, 1–28. CRC Press.

[fsn371254-bib-0064] Kalpana, V. N. S. , J. Mary , S. Mini , N. P. P. Soumya , and S. Mondal . 2021. “ *Hibiscus rosa Sinensis* L. Anthocyanins Prevent Lipid Peroxidation and Improve Antioxidant Status in the Liver of Streptozotocin‐Induced Diabetic Rats.” Bioactive Compounds in Health and Disease 4, no. 10: 240–255.

[fsn371254-bib-0065] Kandhare, A. D. , K. S. Raygude , P. Ghosh , et al. 2012. “Effect of Hydroalcoholic Extract of Hibiscus rosa Sinensis Linn. Leaves in Experimental Colitis in Rats.” Asian Pacific Journal of Tropical Biomedicine 2, no. 5: 337–344. 23569927 10.1016/S2221-1691(12)60053-7 PMC3609304

[fsn371254-bib-0066] Kareem, S. H. , F. N. A. Alhady , and A. Mansour . 2022. “Effect of Hibiscus Rosa Nanoparticles on Sperm Parameters of Male Albino Rats.” International Journal of Health Sciences 6, no. S6: 11017–11028.

[fsn371254-bib-0067] Karnwal, A. 2022. “In Vitro Antibacterial Activity of Hibiscus rosa Sinensis, Chrysanthemum Indicum, and *Calendula officinalis* Flower Extracts Against Gram Negative and Gram Positive Food Poisoning bacteria.” Advances in Traditional Medicine 22, no. 3: 607–619.

[fsn371254-bib-0068] Keservani, R. K. , B. T. Tung , R. K. Kesharwani , and E. D. Ahire . 2024. Plant Metabolites and Vegetables as Nutraceuticals. CRC Press.

[fsn371254-bib-0069] Khadasare, P. M. , S. A. Shinde , S. S. Londe , S. A. Inamdar , and S. J. Kharat . 2024. “Formulation & Evaluation of Hair Growth Serum From Hibiscus Flowers and Leaves.” International Journal of Therapeutic Innovations 2, no. 5: 203–211.

[fsn371254-bib-0070] Khalid, L. , G. H. Rizwani , V. Sultana , H. Zahid , R. Khursheed , and H. Shareef . 2014. “Antidepressant Activity of Ethanolic Extract of Hibiscus rosa Sinenesis Linn.” Pakistan Journal of Pharmaceutical Sciences 27: 5. 25176367

[fsn371254-bib-0071] Khan, I. M. , R. Rahman , A. Mushtaq , and M. Rezgui . 2017. “ *Hibiscus rosa‐sinensis* L.(Malvaceae): Distribution, Chemistry and Uses.” International Journal of Chemical And Biochemical Sciences 12: 147–151.

[fsn371254-bib-0072] Khan, Z. A. , S. A. Naqvi , A. Mukhtar , et al. 2014. “Antioxidant and Antibacterial Activities of *Hibiscus Rosa‐sinensis* Linn Flower Extracts.” Pakistan Journal of Pharmaceutical Sciences 27, no. 3: 469–474. 24811803

[fsn371254-bib-0073] Khandelwal, V. K. M. , R. Balaraman , D. Pancza , and T. Ravingerová . 2011. “Hemidesmus Indicus and *Hibiscus rosa‐sinensis* Affect Ischemia Reperfusion Injury in Isolated Rat Hearts.” Evidence‐Based Complementary and Alternative Medicine 2011, no. 1: 802937. 20953394 10.1155/2011/802937 PMC2952330

[fsn371254-bib-0074] Kumar, A. K. 2020. “Hepatoprotective Activity of Whole Flower of *Hibiscus Rosa‐sinensis* Linn Extracts in Wistar Rats.” Indian Drugs 57, no. 5: 65.

[fsn371254-bib-0075] Kumar Dwivedi, A. , and A. Jain . 2023. “Antioxidant and Antibacterial Activity of Leaves of *Hibiscus rosa‐sinensis* .” International Journal of Phytology Research 3, no. 4: 12–15.

[fsn371254-bib-0076] Kumar, V. , F. Mahdi , A. K. Khanna , et al. 2013. “Antidyslipidemic and Antioxidant Activities of Hibiscus rosa Sinensis Root Extract in Alloxan Induced Diabetic Rats.” Indian Journal of Clinical Biochemistry 28: 46–50. 24381420 10.1007/s12291-012-0223-x PMC3547440

[fsn371254-bib-0077] Lailiyah, M. 2023. “Hair Growth Cream Formulation From Shoe Flower Leaf Ethanol Extract ( *Hibiscus rosa‐sinensis* L.) as a Hair Grower in Rabbit ( *Oryctolagus cuniculus* ).” Jurnal Eduhealth 14, no. 02: 720–728.

[fsn371254-bib-0078] Lima, M. T. N. S. 2022. Evaluation of the Bioactivity of Hibiscus Extracts (Hibiscus Sabdariffa and *H. rosa‐sinensis* ) and Anti‐Inflammatory Potential.

[fsn371254-bib-0079] Lingesh, A. M. P. K. , D. Paul , V. G. M. Naidu , and N. Satheeshkumar . 2019. “AMPK Activating and Anti Adipogenic Potential of *Hibiscus rosa Sinensis* Flower in 3T3‐L1 Cells.” Journal of Ethnopharmacology 233: 123–130. 30593890 10.1016/j.jep.2018.12.039

[fsn371254-bib-0080] Lu, L. , Z. Zhuang , M. Fan , et al. 2022. “Green Formulation of ag Nanoparticles by *Hibiscus rosa‐sinensis* : Introducing a Novel Chemotherapeutic Drug for the Treatment of Liver Cancer.” Arabian Journal of Chemistry 15, no. 2: 103602.

[fsn371254-bib-0081] Magalakshmi, P. , A. Mandhurya , N. Nandhini , C. Abinaya , and J. S. Savithri . 2022. “Antibacterial Activity and Qualitative Phytochemical Study of *Hibiscus rosa Sinensis* .” International Journal of Botany Studies 7, no. 3: 235–239.

[fsn371254-bib-0082] Magdalita, P. M. , and A. O. San Pascual . 2022. “Hibiscus ( *Hibiscus rosa‐sinensis* ): Importance and Classification.” In Floriculture and Ornamental Plants, 483–522. Springer Nature Singapore.

[fsn371254-bib-0083] Mahdy, A. K. , E. Lokes , V. Schöpfel , et al. 2024. “Bulk T Cell Repertoire Sequencing (TCR‐Seq) is a Powerful Technology for Understanding Inflammation‐Mediated Diseases.” Journal of Autoimmunity 149: 103337. 39571301 10.1016/j.jaut.2024.103337

[fsn371254-bib-0084] Mak, Y. W. , L. O. Chuah , R. Ahmad , and R. Bhat . 2013. “Antioxidant and Antibacterial Activities of Hibiscus ( *Hibiscus rosa‐sinensis* L.) and Cassia ( *Senna bicapsularis* L.) Flower Extracts.” Journal of King Saud University, Science 25, no. 4: 275–282.

[fsn371254-bib-0085] Mamun, A. , S. Islam , A. K. Alam , M. A. A. Rahman , and M. Rashid . 2013. “Effects of Ethanolic Extract of *Hibiscus rosa‐sinensis* Leaves on Alloxan‐Induced Diabetes With Dyslipidemia in Rats.” Bangladesh Pharmaceutical Journal 16, no. 1: 27–31.

[fsn371254-bib-0086] Meena, A. K. , A. Jain , K. Pandey , and R. K. Singh . 2014. “Acute Toxicity and Genotoxic Activity of *Hibiscus rosa‐sinensis* Flower Extract.” American Journal of Phytomedicine and Clinical Therapeutics 2, no. 4: 524–529.

[fsn371254-bib-0087] Mejía, J. J. , L. J. Sierra , J. G. Ceballos , J. R. Martínez , and E. E. Stashenko . 2023. “Color, Antioxidant Capacity and Flavonoid Composition in *Hibiscus rosa‐sinensis* Cultivars.” Molecules 28, no. 4: 1779. 36838766 10.3390/molecules28041779 PMC9960340

[fsn371254-bib-0088] Mohan, M. , A. Shinde , and B. Khade . 2011. “Effect of Anthocyanidin Fraction of *Hibiscus Rosa‐sinensis* on Blood Pressure in Deoxycorticosterone Acetate (DOCA)‐Salt‐Hypertensive Rats.” Pharmacology 3: 1097–1111.

[fsn371254-bib-0089] Mohana, P. , A. Singh , F. Rashid , et al. 2024. “Inhibition of Virulence Associated Traits by β‐Sitosterol Isolated From *Hibiscus rosa‐sinensis* Flowers Against *Candida albicans*: Mechanistic Insight and Molecular Docking Studies.” Journal of Microbiology 62: 1–11. 39503955 10.1007/s12275-024-00174-5

[fsn371254-bib-0090] Moyo, H. N. 2024. “The Impact of Food Processing Techniques on Nutrient Retention and Bioavailability.” Iconic Research and Engineering Journals 8, no. 2: 435–460.

[fsn371254-bib-0091] Mustaffa, F. , S. Parasuraman , and G. Sahgal . 2020. “Wound Healing Activity of Herbal Ointment Containing the Extracts of *Hibiscus rosa‐sinensis* Flowers and *Curcuma longa* Rhizomes.” Free Radicals and Antioxidants 10, no. 2: 86–88.

[fsn371254-bib-0092] Nade, V. S. , S. Dwivedi , L. A. Kawale , C. D. Upasani , and A. V. Yadav . 2009. “Effect of Hibiscus rosa Sinensis on Reserpine‐Induced Neurobehavioral and Biochemical Alterations in Rats.” Indian Journal of Experimental Biology 47, no. 7: 559–563. 19761039

[fsn371254-bib-0093] Nade, V. S. , L. A. Kawale , S. Dwivedi , and A. V. Yadav . 2010. “Neuroprotective Effect of Hibiscus rosa Sinensis in an Oxidative Stress Model of Cerebral Post‐Ischemic Reperfusion Injury in Rats.” Pharmaceutical Biology 48, no. 7: 822–827. 20645783 10.3109/13880200903283699

[fsn371254-bib-0094] Nagar, N. V. 2012. “Antimicrobial Evaluation of *hibiscus rosa‐sinensis* Plant Extracts Against Some Pathogenic Bacteria.” Bulletin of Environmental and Scientific Research 1, no. 3–4: 14–17.

[fsn371254-bib-0095] Nagarajappa, R. , M. Batra , A. J. Sharda , et al. 2015. “Antimicrobial Effect of *Jasminum grandiflorum* L. and *Hibiscus rosa‐sinensis* L. Extracts Against Pathogenic Oral Microorganisms‐An In Vitro Comparative Study.” Oral Health & Preventive Dentistry 13, no. 4: 341–348. 24046822 10.3290/j.ohpd.a30601

[fsn371254-bib-0096] Nath, P. , and A. K. Yadav . 2014. “Acute and Sub‐Acute Oral Toxicity Assessment of the Methanolic Extract From Leaves of *Hibiscus rosa‐sinensis* L in Mice.” Journal of Intercultural Ethnopharmacology 4: 70. 26401388 10.5455/jice.20141028021746 PMC4566762

[fsn371254-bib-0097] Nayak, D. , S. Ashe , P. R. Rauta , and B. Nayak . 2015. “Biosynthesis, Characterisation and Antimicrobial Activity of Silver Nanoparticles Using *Hibiscus rosa‐sinensis* Petals Extracts.” IET Nanobiotechnology 9, no. 5: 288–293. 26435282 10.1049/iet-nbt.2014.0047

[fsn371254-bib-0098] Ngan, L. T. M. , M. T. Tan , N. V. M. Hoang , et al. 2021. “Antibacterial Activity of *Hibiscus rosa‐sinensis* L. Red Flower Against Antibiotic‐Resistant Strains of Helicobacter Pylori and Identification of the Flower Constituents.” Brazilian Journal of Medical and Biological Research 54: e10889. 34008759 10.1590/1414-431X2020e10889 PMC8130102

[fsn371254-bib-0099] Noman, A. M. , M. T. Sultan , S. Zafar , et al. 2025. “Thymol and Carvacrol: Molecular Mechanisms, Therapeutic Potential, and Synergy With Conventional Therapies in Cancer Management.” Food Science & Nutrition 13, no. 9: e70936. 40964147 10.1002/fsn3.70936 PMC12436185

[fsn371254-bib-0100] Nwibo, D. D. , M. I. Eze , and T. M. Okonkwo . 2016. “Effects of *Hibiscus rosa‐sinensis* Leaf Products on Haematological Indices, Lipid Profile and Hepatic Parameters of Hyperlipidemic Rat.” African Journal of Pharmacy and Pharmacology 10, no. 12: 223–229.

[fsn371254-bib-0101] Oluwamodupe, C. , A. O. Adeleye , O. O. Babalola , and P. O. Ottu . 2024. Investigation of Proinflammatory Genes Expression in STZ‐Induced Diabetic Rats Treated With Extract of *Hibiscus rosa‐sinensis* Flower.

[fsn371254-bib-0102] Parihar, A. , B. K. Dubey , D. K. Basedia , M. K. Patel , and S. Shah . 2024. “Development and Characterization of Hair Oil for Control Hair Fall and Stimulating Hair Growth.” International Journal of Pharmacy & Life Sciences 15, no. 12: 41.

[fsn371254-bib-0103] Patrice, A. R. J. B. A. , L. Cabuso , and C. M. Narvacan . 2017. “Antibacterial Activity of Ethanolic Extract of *Hibiscus rosa‐sinensis* Flower Against Staphylococcus Epidermidis and *staphylococcus saprophyticus* . Lyceum of the Philippines‐St.” Cabrini College of Allied Medicine 2, no. 2: 39–50.

[fsn371254-bib-0104] Pethe, M. , S. Yelwatkar , V. Gujar , S. Varma , and S. Manchalwar . 2017. “Antidiabetic, Hypolipidimic and Antioxidant Activities of *Hibiscus rosa Sinensis* Flower Extract in Alloxan Induced Diabetes in Rabbits.” International Journal of Biomedical and Advance Research 8: 138–143.

[fsn371254-bib-0105] Pethe, M. , S. Yelwatkar , S. Manchalwar , and V. Gujar . 2017. “Evaluation of Biological Effects of Hydroalcoholic Extract of *Hibiscus rosa Sinensis* Flowers on Alloxan Induced Diabetes in Rats.” Drug Research 67, no. 08: 485–492. 28521371 10.1055/s-0043-109434

[fsn371254-bib-0106] Pieracci, Y. , L. Pistelli , M. Lari , et al. 2021. “ *Hibiscus rosa‐sinensis* as Flavoring Agent for Alcoholic Beverages.” Applied Sciences 11, no. 21: 9864.

[fsn371254-bib-0107] Pillai, S. S. , and S. Mini . 2018. “Attenuation of High Glucose Induced Apoptotic and Inflammatory Signaling Pathways in RIN‐m5F Pancreatic β Cell Lines by Hibiscus rosa Sinensis L. Petals and Its Phytoconstituents.” Journal of Ethnopharmacology 227: 8–17. 30120944 10.1016/j.jep.2018.08.022

[fsn371254-bib-0108] Priya, K. , and H. P. Sharma . 2021. “Phytochemical Analysis and Antimicrobial Activity of *Hibiscus rosa Sinensis* .” European Journal of Biotechnology and Bioscience 9, no. 1: 21–26.

[fsn371254-bib-0109] Raduan, S. Z. , M. W. H. Abdul Aziz , A. H. Roslida , Z. A. Zakaria , A. Zuraini , and M. N. Hakim 2013. “Anti‐Inflammatory Effects of *Hibiscus rosa‐sinensis* L. and *Hibiscus rosa‐sinensis* Var. Alba Ethanol Extracts.” International Journal of Pharmacy and Pharmaceutical Sciences 5, no. 4: 754–762.

[fsn371254-bib-0110] Rajavelu, I. , and R. Bs . 2024. “Apoptosis Induction by Plectranthus Amboinicus and *Hibiscus rosa‐sinensis* Extracts in HepG2 Cells: Insights Into Cytotoxicity and Gene Regulation.”

[fsn371254-bib-0111] Rajendran, A. , E. Siva , C. Dhanraj , and S. Senthilkumar . 2018. “A Green and Facile Approach for the Synthesis Copper Oxide Nanoparticles Using *Hibiscus rosa‐sinensis* Flower Extracts and It's Antibacterial Activities.” Journal of Bioprocessing and Biotechniques 8, no. 3: 324.

[fsn371254-bib-0112] Rakhi, A. R. , I. S. Jacob , S. Juno , K. R. Nair , S. Pooja , and A. K. Anjana . 2022. “Pathological and Pharmacological Review of Liver.” World Journal of Pharmaceutical Research 11, no. 4: 1943–1955.

[fsn371254-bib-0113] Ralte, L. , H. Sailo , and Y. T. Singh . 2024. “Ethnobotanical Study of Medicinal Plants Used by the Indigenous Community of the Western Region of Mizoram, India.” Journal of Ethnobiology and Ethnomedicine 20, no. 1: 2. 38172927 10.1186/s13002-023-00642-z PMC10765666

[fsn371254-bib-0114] Rassem, H. H. , M. H. B. Khamidun , U. F. M. Ali , T. Hadibarata , and N. A. Alrabie . 2024. “Comprehensive Analysis of Antioxidant and Antibacterial Activities of Water and Methanol Extracts of Hibiscus Flower.” Journal of King Saud University, Science 36, no. 11: 103506.

[fsn371254-bib-0115] Raza, H. , M. T. Sultan , S. Khalid , et al. 2025. “Ultrasound‐Assisted Extraction of *Hibiscus rosa‐sinensis* Extracts for the Development of Functional Tea.” Ultrasonics Sonochemistry 122: 107628. 41138490 10.1016/j.ultsonch.2025.107628 PMC12594926

[fsn371254-bib-0116] Rehana, D. , D. Mahendiran , R. S. Kumar , and A. K. Rahiman . 2017. “Evaluation of Antioxidant and Anticancer Activity of Copper Oxide Nanoparticles Synthesized Using Medicinally Important Plant Extracts.” Biomedicine & Pharmacotherapy 89: 1067–1077. 28292015 10.1016/j.biopha.2017.02.101

[fsn371254-bib-0117] Rehman, M. U. , B. Nisar , A. M. Yatoo , et al. 2024. “After Effects of Pharmaceuticals and Personal Care Products (PPCPs) on the Biosphere and Their Counteractive Ways.” Separation and Purification Technology 342: 126921.

[fsn371254-bib-0118] Rengarajan, S. , V. Melanathuru , C. Govindasamy , V. Chinnadurai , and M. F. Elsadek . 2020. “Antioxidant Activity of Flavonoid Compounds Isolated From the Petals of Hibiscus rosa Sinensis.” Journal of King Saud University, Science 32, no. 3: 2236–2242.

[fsn371254-bib-0119] Rose, L. C. , N. N. S. Rusdi , A. Asari , M. E. Abd Wahid , and H. Suhaimi . 2020. “Potential Hair Growth of Crude Extract From *Hibiscus rosa‐sinensis* Linn.” Archives of Pharmacy Practice 11, no. 4–2020: 13–19.

[fsn371254-bib-0120] Ruban, P. , and K. Gajalakshmi . 2012. “In Vitro Antibacterial Activity of Hibiscus rosa–Sinensis Flower Extract Against Human Pathogens.” Asian Pacific Journal of Tropical Biomedicine 2, no. 5: 399–403. 23569938 10.1016/S2221-1691(12)60064-1 PMC3609315

[fsn371254-bib-0121] Sachdewa, A. , and L. D. Khemani . 2003. “Effect of Hibiscus rosa Sinensis Linn. Ethanol Flower Extract on Blood Glucose and Lipid Profile in Streptozotocin Induced Diabetes in Rats.” Journal of Ethnopharmacology 89, no. 1: 61–66. 14522433 10.1016/s0378-8741(03)00230-7

[fsn371254-bib-0122] Sachdewa, A. , R. Nigam , and L. D. Khemani . 2001. “Hypoglycemic Effect of Hibiscus rosa Sinensis L. Leaf Extract in Glucose and Streptozotocin Induced Hyperglycemic Rats.” Indian Journal of Experimental Biology 39, no. 3: 284–286. 11495291

[fsn371254-bib-0123] Sağlam, A. , M. A. Özüsağlam , and I. Çelik . 2023. “Determination of Antimicrobial Activity of Cream Formulation Developed With *Hibiscus rosa‐sinensis* Extract and Probiotic.” Manas Journal of Agriculture, Veterinary and Life Sciences 13, no. 2: 126–132.

[fsn371254-bib-0124] Sahu, C. R. 2016. “Mechanisms Involved in Toxicity of Liver Caused by Piroxicam in Mice and Protective Effects of Leaf Extract of *Hibiscus rosa‐sinensis* L.” Clinical Medicine Insights: Arthritis and Musculoskeletal Disorders 9: S29463. 10.4137/CMAMD.S29463 PMC4720181 26819562

[fsn371254-bib-0125] Sanadheera, S. , D. Subasinghe , M. N. Solangaarachchi , M. Suraweera , N. Y. Suraweera , and N. Tharangika . 2021. “ *Hibiscus rosa‐sinensis* L.(Red Hibiscus) Tea, Can It be Used as A Home‐Remedy to Control Diabetes and Hypercholesterolemia?” Biology, Medicine, & Natural Product Chemistry 10, no. 1: 59–65.

[fsn371254-bib-0126] Sattar Ali, Z. 2022. “Hepatic Impact of Different Concentrations of Hibiscus Rosa Zinc Oxide Nanoparticles on Rats.” Archives of Razi Institute 77, no. 3: 1199–1206. 36618294 10.22092/ARI.2022.357530.2060 PMC9759250

[fsn371254-bib-0127] Sawarkar, A. , C. R. Jangde , P. D. Thakre , R. Kadoo , and S. Shelu . 2009. “Analgesic Activity of Hibiscus rosa Sinensis Linn in Rat.” Veterinary World 2: 353–354.

[fsn371254-bib-0128] Seyyednejad, S. M. , H. Koochak , E. Darabpour , and H. Motamedi . 2010. “A Survey on Hibiscus rosa—Sinensis, *Alcea rosea* L. and *Malva neglecta* Wallr as Antibacterial Agents.” Asian Pacific Journal of Tropical Medicine 3, no. 5: 351–355.

[fsn371254-bib-0129] Shafiq, N. , F. Yasmin , S. Noreen , A. Shahzad , M. Rashid , and M. Bilal . 2021. “Phytochemical Profiling of Medicinal Plants Extracts and Their Antioxidant and Anticancer Potentialities Against Human Liver cancer (Hep g2) Cell Lines.” Revista de Chimimie 72, no. 2: 1.

[fsn371254-bib-0130] Shah, F. , and E. Wu . 2019. “Soil and Crop Management Strategies to Ensure Higher Crop Productivity Within Sustainable Environments.” Sustainability 11, no. 5: 1485.

[fsn371254-bib-0131] Shaheen, S. , S. Khalid , R. Siqqique , et al. 2023. “Comparative Taxonomical, Biological and Pharmacological Potential of Healthy and Geminivirus Infected Leaves of *Hibiscus rosa‐sinensis* L.: First Report.” Microbial Pathogenesis 185: 106428. 37977480 10.1016/j.micpath.2023.106428

[fsn371254-bib-0132] Sharma, K. R. , and K. Thakur . 2022. “Determination of Phenolic and Flavonoid Content, Antioxidant and α‐Amylase Inhibitory Activity of Leaf and Flower Extracts of Clerodendrum Infortunatum and *Hibiscus rosa Sinensis* Growing in Bara Nepal.” Journal of Balkumari College 11, no. 1: 20–26.

[fsn371254-bib-0133] Sharma, P. , A. Rajput , N. Kumar , et al. 2023. “Antioxidant Activity and Inhibitory Potential of *Hibiscus rosa‐sinensis* Flower Extract Against the Key Enzymes Relevant for Hyperglycemia: In‐Vitro and In‐Silico Studies.” Minerva Biotechnology & Biomolecular 35, no. 2: 90.

[fsn371254-bib-0134] Sheikhar, C. , R. Rani , A. P. Singh , and A. P. Singh . 2024. “Evaluation of Anti‐Depressant Activity of Hibiscus rosa Sinensis Leaves in Mice.” Journal of Drug Delivery & Therapeutics 14, no. 12: 38.

[fsn371254-bib-0135] Shen, H. , Y. Zheng , R. Chen , X. Huang , and G. Shi . 2021. “Neuroprotective Effects of Quercetin 3‐O‐Sophoroside From *Hibiscus rosa‐sinensis* Linn. On Scopolamine‐Induced Amnesia in Mice.” Journal of Functional Foods 76: 104291.

[fsn371254-bib-0136] Shewale, P. B. , R. A. Patil , and Y. A. Hiray . 2012. “Antidepressant‐Like Activity of Anthocyanidins From *Hibiscus rosa‐sinensis* Flowers in Tail Suspension Test and Forced Swim Test.” Indian Journal of Pharmacology 44, no. 4: 454–457. 23087504 10.4103/0253-7613.99303 PMC3469946

[fsn371254-bib-0137] Shi, B. , H. Wang , A. Nawaz , et al. 2024. “Dual Functional Roles of Nutritional Additives in Nutritional Fortification and Safety of Thermally Processed Food: Potential, Limitations, and Perspectives.” Comprehensive Reviews in Food Science and Food Safety 23, no. 1: e13268. 38284588 10.1111/1541-4337.13268

[fsn371254-bib-0138] Sidhu, R. , S. Kaushal , V. Kaur , and P. Sharma . 2023. “Chemical Composition, Synergistic Antimicrobial and Antioxidant Potential of *Hibiscus rosa‐sinensis* L. Leaves Essential Oil and Its Major Compound.” Journal of Essential Oil Bearing Plants 26, no. 2: 469–485.

[fsn371254-bib-0139] Sikarwar Mukesh, S. , and M. B. Patil . 2011. “Antihyperlipidemic Effect of Ethanolic Extract of *Hibiscus rosa Sinensis* Flowers in Hyperlipidemic Rats.” RGUHS Journal of Pharmaceutical Sciences 1, no. 2: 117–122.

[fsn371254-bib-0140] Silva, T. L. , L. A. F. Afiune , R. Q. de Moraes Souza , et al. 2015. “Effect of *Hibiscus rosa Sinensis* Aqueous Extract Treatment on Biochemical Parameters in Diabetic Pregnant Rats.” Diabetology & Metabolic Syndrome 7: 1.25810781

[fsn371254-bib-0141] Singaravelan, K. , G. Krishnamoorthi , V. Jeevanantham , and V. P. Muralidharan . 2024. “Antibacterial and Methylene Blue Dye Degradation Activity of *Hibiscus rosa Sinensis* Synthesized CoFe2O4 Nanocomposites.” ChemistrySelect 9, no. 41: e202402697.

[fsn371254-bib-0142] Singh, K. G. , S. Sonia , and N. Konsoor . 2018. “In‐Vitro and Ex‐Vivo Studies on the Antioxidant, Anti‐Inflammatory and Antiarthritic Properties of *Camellia sinensis* , *Hibiscus Rosa Sinensis*, *Matricaria chamomilla* , Rosa SP., Zingiber Officinale tea Extracts.” Inflammation 49: 50.

[fsn371254-bib-0143] Sinha, A. , and O. A. Asimi . 2007. “China Rose (Hibiscus Rosasinensis) Petals: A Potent Natural Carotenoid Source for Goldfish ( *Carassius auratus* L.).” Aquaculture Research 38, no. 11: 1123–1128.

[fsn371254-bib-0144] Sivaraman, C. M. , and F. Saju . 2021. “Medicinal Value of *Hibiscus rosa Sinensis*: A Review.” International Journal of Pharmacognosy and Chemistry 2, no. 1: 1–11.

[fsn371254-bib-0145] Somchit, M. N. , A. Zuraini , W. P. Tan , and M. S. Cheema . 2008. “Potent Inhibition of Prostaglandin D2 Induced Inflammation by *Hibiscus rosa‐sinensis* Crude Extract.” Planta Medica 74: PA41.

[fsn371254-bib-0146] Soni, D. , and A. Gupta . 2011. “An Evaluation of Antipyretic and Analgesic Potentials of Aqueous Root Extract of *Hibiscus Rosa Sinesis* Linn. (Malvacae).” International Journal of Research in Phytochemistry and Pharmacology 1, no. 3: 184–186.

[fsn371254-bib-0147] Sruthi, T. , C. K. Rao , R. Z. Michael , S. M. Nissy , and D. S. Prakash . 2021. “In‐Vitro Anti‐Inflammatory and Anti‐Arthritic Activity of Ethanolic Extract of *Hibiscus Rosa Sinensis* Leaves.” Rasayan Journal of Chemistry 14, no. 4: 2279–2284.

[fsn371254-bib-0148] Sucharitha, M. , and M. Nagamani . 2021. “Evaluation of Anti‐Depressant Property of Hibiscus Rosasinensis Plant Extracts.” International Journal of Pharma and Bio Sciences 11, no. 3: 97–103.

[fsn371254-bib-0149] Surya, S. , G. D. Kumar , and R. Rajakumar . 2016. “Green Synthesis of Silver Nanoparticles From Flower Extract of *Hibiscus Rosa*‐*Sinensis* and Its Antibacterial Activity.” International Journal of Innovative Research in Science, Engineering and Technology 5, no. 4: 5242–5247.

[fsn371254-bib-0150] Tawfeeq, A. A. , T. A. Tawfeeq , I. S. Abaas , and S. H. Salah . 2024. “Mini Review: Phytochemistry and Pharmacological Activity of *Hibiscus Rosa‐sinensis* Plant.” Protein: Jurnal Ilmu Keperawatan Dan Kebidanan 2, no. 4: 95–101.

[fsn371254-bib-0151] Thi, H. N. , T. P. Anh , H. N. Thanh , et al. 2021. “Study on the Antioxidant Capacity of *Hibiscus rosa‐sinensis* Decoction In Vivo in *Mus musculus* Var. Albino.” Nauchnye Rezul'taty Biomeditsinskikh Issledovaniĭ 7, no. 2: 149–155.

[fsn371254-bib-0152] Tong, C. Y. , and T. S. Tee . 2022. “Antioxidant and Antibacterial Activities of Flower and Leaf Extracts of Hybrid, White and Orange Hibiscus (*Hibiscus Rosa‐sinensis*).” Strengthening Regional Collaboration in Science & Technology 112.

[fsn371254-bib-0153] Tyagi, S. , A. Kumar , and P. K. Tyagi . 2017. “Comparative Analysis of Metal Nanoparticles Synthesized From *Hibiscus rosa Sinesis* and Their Antibacterial Activity Estimation Against Nine Pathogenic bacteria.” Asian Journal of Pharmaceutical and Clinical Research 10, no. 5: 323–329.

[fsn371254-bib-0154] Uddin, B. , T. Hossan , S. Paul , T. Ahmed , T. Nahar , and S. Ahmed . 2010. “Antibacterial Activity of the Ethanol Extracts of *Hibiscus rosa‐sinensis* Leaves and Flowers Against Clinical Isolates of bacteria.” Bangladesh Journal of Life Sciences 22, no. 2: 65–73.

[fsn371254-bib-0155] Udo, I. J. , M. G. Ben , C. U. Etuk , and A. I. Tiomthy . 2016. “Phytochemical, Proximate and Antibacterial Properties of *Hibiscus rosa‐sinensis* L. Leaf.” Journal of Medicinal Plants Studies 4, no. 5: 193–195.

[fsn371254-bib-0156] Umar, H. B. , S. Abdulkadir , B. Ibrahim , and H. Ibrahim . 2024. “Phytochemical Screening, Acute Toxicity and Antibacterial Activity of Methanolic Stem Bark Extract of *Hibiscus Rosa‐sinensis* .” International Journal of Emerging Multidisciplinaries: Biomedical and Clinical Research 2, no. 1: 1–18.

[fsn371254-bib-0157] Uzombah, T. A. 2022. “The Implications of Replacing Synthetic Antioxidants With Natural Ones in the Food Systems.” In Natural Food Additives. IntechOpen.

[fsn371254-bib-0158] Valdivié, M. , and Y. Martínez . 2022. “ *Hibiscus rosa‐sinensis* Forage as a Potential Feed for Animals: A Review.” Animals 12, no. 3: 288. 35158612 10.3390/ani12030288 PMC8833687

[fsn371254-bib-0159] Vankar, P. S. , and D. Shukla . 2011. “Natural Dyeing With Anthocyanins From *Hibiscus rosa Sinensis* Flowers.” Journal of Applied Polymer Science 122, no. 5: 3361–3368.

[fsn371254-bib-0160] Vankar, P. S. , and J. Srivastava . 2008. “Comparative Study of Total Phenol, Flavonoid Contents and Antioxidant Activity in Canna Indica and *Hibiscus rosa Sinensis*: Prospective Natural Food Dyes.” International Journal of Food Engineering 4, no. 3: 1–16.

[fsn371254-bib-0161] Vasudeva, N. , and S. K. Sharma . 2008. “Post‐Coital Antifertility Activity of *Hibiscus rosa‐sinensis* Linn. Roots.” Evidence‐Based Complementary and Alternative Medicine 5, no. 1: 91–94. 18317554 10.1093/ecam/nem003 PMC2249740

[fsn371254-bib-0162] Velayutham, L. , C. Parvathiraja , D. C. Anitha , et al. 2022. “Photocatalytic and Antibacterial Activity of CoFe2O4 Nanoparticles From *Hibiscus rosa‐sinensis* Plant Extract.” Nanomaterials 12, no. 20: 3668. 36296858 10.3390/nano12203668 PMC9609893

[fsn371254-bib-0163] Velayutham, L. , C. Parvathiraja , D. C. Anitha , et al. 2023. “Antibacterial and Photocatalytic Dye Degradation Activities of Green Synthesized Nise Nanoparticles From *hibiscus rosa‐sinensis* Leaf Extract.” Water 15, no. 7: 1380.

[fsn371254-bib-0164] Vignesh, R. M. , and B. R. Nair . 2018. “A Study on the Antioxidant and Antibacterial Potential of the Mucilage Isolated From *Hibiscus rosa‐sinensis* Linn.(Malvaceae).” Journal of Pharmacognosy and Phytochemistry 7, no. 2: 1633–1637.

[fsn371254-bib-0165] Vijayanand, P. , V. Jyothi , N. Aditya , and A. Mounika . 2018. “Development and Characterization of Solid Lipid Nanoparticles Containing Herbal Extract: In Vivo Antidepressant Activity.” Journal of Drug Delivery 2018, no. 1: 2908626. 29973993 10.1155/2018/2908626 PMC6008679

[fsn371254-bib-0166] Vijayaraj, R. , and N. S. Kumaran . 2017. “Biosynthesis of Silver Nanoparticles From *Hibiscus rosa Sinensis*: An Approach Towards Animicrobial Activity on Fish Pathogen *aeromonas hydrophila* .” International Journal of Pharmaceutical Sciences and Research 8, no. 1: 5241–5246.

[fsn371254-bib-0167] Weerasingha, W. M. R. K. S. , M. H. T. K. Chandrasiri , K. H. I. K. Hewawitharana , and J. K. H. Sampath . 2021. “Evaluation of Anthocyanin Extracted From Hibiscus Rosa Synesis as a Natural Food Colorant. Data‐Driven Scientific Research for Sustainable.” Innovations 10: 17–20.

[fsn371254-bib-0168] Yadav, N. , S. Singh Chandel , T. Venkatachalam , and S. N. Fathima . 2024. “Herbal Medicine Formulation, Standardization, and Commercialization Challenges and Sustainable Strategies for Improvement.” In Herbal Medicine Phytochemistry: Applications and Trends, 1769–1795. Springer International Publishing.

[fsn371254-bib-0169] Yahaya, N. A. , N. K. Anuar , and N. M. Saidin . 2023. “ *Hibiscus rosa‐sinensis* Mucilage as a Functional Polymer in Pharmaceutical Applications: A Review.” International Journal of Applied Pharmaceutics 15, no. 1: 44–49.

[fsn371254-bib-0170] Yang, X. , G. N. Rajivgandhi , G. Ramachandran , et al. 2020. “Preparative HPLC Fraction of *Hibiscus rosa‐sinensis* Essential Oil Against Biofilm Forming *Klebsiella pneumoniae* .” Saudi Journal of Biological Sciences 27, no. 10: 2853–2862. 32994746 10.1016/j.sjbs.2020.07.008 PMC7499370

[fsn371254-bib-0171] Yasin, Z. , M. R. Khan , M. A. Shabbir , and B. Israr . 2025. “Exploring the Therapeutic Potential of Matricaria Chamomilla and *Hibiscus Rosa‐sinensis* Against Diabetes Mellitus.” Pakistan Veterinary Journal 45, no. 2: 759–766.

[fsn371254-bib-0172] Yu, M. G. , D. Gordin , J. Fu , K. Park , Q. Li , and G. L. King . 2024. “Protective Factors and the Pathogenesis of Complications in Diabetes.” Endocrine Reviews 45, no. 2: 227–252. 37638875 10.1210/endrev/bnad030 PMC10911956

[fsn371254-bib-0173] Zaki, L. H. , S. M. Mohamed , S. A. Bashandy , F. A. Morsy , K. M. Tawfik , and A. A. Shahat . 2017. “Hypoglycemic and Antioxidant Effects of *Hibiscus rosa‐sinensis* L. Leaves Extract on Liver and Kidney Damage in Streptozotocin Induced Diabetic Rats.” African Journal of Pharmacy and Pharmacology 11, no. 13: 161–169.

[fsn371254-bib-0174] Zemede, J. , T. Mekuria , C. O. Ochieng , G. E. Onjalalaina , and G. W. Hu . 2024. “Ethnobotanical Study of Traditional Medicinal Plants Used by the Local Gamo People in Boreda Abaya District, Gamo Zone, Southern Ethiopia.” Journal of Ethnobiology and Ethnomedicine 20, no. 1: 28. 38419092 10.1186/s13002-024-00666-z PMC10900619

[fsn371254-bib-0175] Zuhaira, S. , S. Naz , and P. M. Ridzuan . 2020. “The Efficacy of *Hibiscus rosa‐sinensis* Leaf Extracts Against candida SPP, Causing Candidiasis.” Journal of Science and Mathematics Letters 8, no. 1: 1–5.

[fsn371254-bib-0176] Zulkurnain, E. I. , S. Ramli , A. A. Ali , R. J. James , I. S. Kamarazaman , and H. Halim . 2023. “The Phytochemical and Pharmacological Effects of *Hibiscus rosa‐sinensis* : A Review.” International Journal of Pharmaceutical Investigation 13, no. 3: 422.

[fsn371254-bib-0177] Zulkurnain, I. H. , M. Sayuti , A. S. Kamaruzaman , et al. 2025. “Synergistic Wound Healing Effects of *Hibiscus rosa‐Sinensis* and *Centella asiatica* Extracts Combination (HRSCA): In Vivo and 3D Organotypic Models.” Journal of Health Science and Medical Research 44, no. 1: 20251214.

